# Integrating Emerging Polymer Chemistries for the Advancement of Recyclable, Biodegradable, and Biocompatible Electronics

**DOI:** 10.1002/advs.202101233

**Published:** 2021-05-20

**Authors:** Jerika A. Chiong, Helen Tran, Yangju Lin, Yu Zheng, Zhenan Bao

**Affiliations:** ^1^ Department of Chemistry Stanford University Stanford CA 94305‐5025 USA; ^2^ Department of Chemistry University of Toronto Toronto ON M5S 3H6 Canada; ^3^ Department of Chemical Engineering Stanford University Stanford CA 94305‐5025 USA

**Keywords:** biocompatible polymers, biodegradable polymers, molecular design, recyclable polymers, sustainable electronics

## Abstract

Through advances in molecular design, understanding of processing parameters, and development of non‐traditional device fabrication techniques, the field of wearable and implantable skin‐inspired devices is rapidly growing interest in the consumer market. Like previous technological advances, economic growth and efficiency is anticipated, as these devices will enable an augmented level of interaction between humans and the environment. However, the parallel growing electronic waste that is yet to be addressed has already left an adverse impact on the environment and human health. Looking forward, it is imperative to develop both human‐ and environmentally‐friendly electronics, which are contingent on emerging recyclable, biodegradable, and biocompatible polymer technologies. This review provides definitions for recyclable, biodegradable, and biocompatible polymers based on reported literature, an overview of the analytical techniques used to characterize mechanical and chemical property changes, and standard policies for real‐life applications. Then, various strategies in designing the next‐generation of polymers to be recyclable, biodegradable, or biocompatible with enhanced functionalities relative to traditional or commercial polymers are discussed. Finally, electronics that exhibit an element of recyclability, biodegradability, or biocompatibility with new molecular design are highlighted with the anticipation of integrating emerging polymer chemistries into future electronic devices.

## Graphical abstract

With the rapid growth of consumer electronics, their harmonious integration into modern society and the environment is contingent on new eco‐ and human‐friendly polymer technologies. This review explores emerging recyclable, biodegradable, and biocompatible polymer chemistries with a focus on molecular design for incorporation into the next‐generation of sustainable electronics with unrealized capabilities.

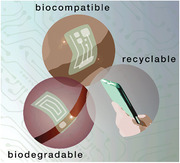

## 1 Introduction

As we become increasingly dependent on consumer plastics and electronics, it is highly desirable for these materials to be seamlessly integrated with the environment and human health. Their harmonious integration into our ecosystem relies on the design of materials to be recyclable, degradable in environmentally relevant conditions, and interfaced with living systems without an adverse impact. Worldwide, plastics approximately account for 150 million tons of solid waste annually, and discarded electronic gadgets additionally constitute 50 million tons of electronic waste (“e‐waste”).^[^ ^1^, ^2^ ^]^ Only 10–20% of these waste products are recycled, with the majority going directly into landfills and incinerators, much of which leach hazardous chemicals and toxic metals into the environment and ecology. While these low percentages for recycling can be partially attributed to consumer habits as well as the difficulty and cost associated with materials separation, they also leave great room for improvement for plastic and electronic industries to design materials that can be recycled or degraded using efficient, cost‐effective techniques or in a wider variety of environments. Electronics that can be recycled or degraded without leaving a negative footprint on the environment would reduce the amount of e‐waste by mitigating the negative effects of improper disposal. Recycling electronic components would conserve scarce natural elements (e.g., gallium, indium) and other valuable resources. Moreover, implantable devices that can degrade under physiological conditions into non‐toxic byproducts or that can function without an adverse immunological response will expand their impact on various biomedical applications. These temporary implants would be adsorbed by the body after a designated time of use, reducing the risk of infection and complications caused by secondary removal procedures. Notably, the integration of wearable and implantable electronics into human life and health is heavily dependent on the biocompatibility of these devices. The advancement of new technologies while promoting environmental and human health and a sustainable future relies on the rapidly growing progress of these recyclable, biodegradable, and biocompatible chemistries and their integration into electronics.

Although most commercial electronics are composed of and reliant on inorganic materials, organic materials possess a wide array of desirable properties for the realization of eco‐ and human‐friendly electronics. Polymers are attractive due to their scalability, solution processability, ability to be rationally tuned by synthetic design, and diverse material properties (e.g., stretchability, toughness, conformability, conductivity). Polymer‐based electronics typically use insulating polymers for various device components (i.e., encapsulants, substrates, dielectrics) and conjugated or doped polymers as the electronically‐active component (i.e., semiconductor, conductor). Much work has been done in designing polymers to be eco‐ and human‐friendly as well as to impart desirable properties (e.g., self‐healing, stimuli‐responsivity, adhesivity) for electronic applications. For example, our group demonstrated fully biodegradable and biocompatible thin film transistors (TFTs) based on a ultrathin cellulose substrate and acid‐labile polymer semiconductors that completely decomposed under mildly acidic (pH = 4.6) conditions within thirty days.^[^ ^3^ ^]^ Furthermore, stretchability was realized when these polymer semiconductors were blended with a biodegradable elastomer.^[^ ^4^ ^]^ These technologies yield broader applications in low‐cost, eco‐friendly, and bio‐integrated organic electronics.

In this review, we discuss how polymers are designed to impart properties that allow them to be recyclable, biodegradable, or biocompatible as well as how these polymers are used in various devices for electronic applications. We recognize that there have been several detailed reviews on recyclable polymers, biodegradable and biocompatible electronics, and bioresorbable electronics.^[^ ^1^, ^2^, ^5^, ^6^, ^7^, ^8^, ^9^, ^10^, ^11^, ^12^ ^]^ Those written on recyclable polymers focus on commercial plastics and do not discuss their potential use in electronics, while the biodegradable and biocompatible electronics reviews mainly examine inorganic electronics or do not cover recent discoveries in biodegradable and biocompatible chemistries. This review aims to focus on the underlying chemistries of sustainable polymers as well as provide prospective into how these new polymer chemistries can be used in electronics.

**Figure 1:**
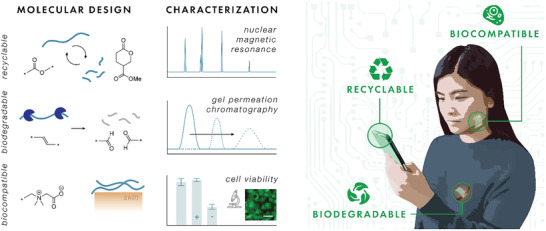
Schematic illustration of this review. The next generation of eco‐ and human‐friendly electronics rely on emerging molecular design and characterization techniques for recyclable, biodegradable, and biocompatible polymers.

First, we give an overview on recyclable, biodegradable, and biocompatible terminology. Subsequently, we examine the current standards and regulations implemented by governing organizations as well as the characterization methods and tools used in studies for each of these polymer categories. For molecular design, we discuss established chemistries for sustainable polymers and then examine emerging polymers in the field and their advantages over existing chemistries. Finally, we review how polymers have been used in recyclable, biodegradable, or biocompatible electronic applications. It is important to note that the components (e.g., substrates, dielectrics, semiconductors, conductors) that make up electronic devices are composed of vastly different polymeric materials. Each component plays a crucial role in the functionality of the device. Currently, there is a gap between the polymeric materials commonly used in electronics and emerging polymer chemistries reported in literature over the past few years. We hope to provide readers with a fundamental understanding of the molecular design required to achieve these eco‐ and human‐friendly emergent properties as well as encourage the synergistic effort to bridge the gap between molecular design and device fabrication. The next‐generation of sustainable electronics with advanced, unrealized capabilities are contingent on the development and integration of emerging polymer chemistries (**Figure** **1**).

## 2 Overview and Classifications

For this review, the terms “recyclable,” “biodegradable,” and “biocompatible” and the associated subcategories are defined based on reported literature.^[^ ^1^, ^5^, ^8^, ^10^, ^13^, ^14^ ^]^ Recyclable, biodegradable, and biocompatible polymers have been widely studied from both a chemistry and engineering perspective for applications in sustainable plastics and electronics. Discrepancies between the various definitions of these terms have evolved from the numerous studies from different disciplines that have emerged over the years. To clarify these discrepancies, classifications and definitions of each class of material are described based on terms that are generally used and accepted by researchers in both fields.

### 2.1 Recyclable

Recyclable polymers have been well classified in terms of recyclable, commercial plastics, whose terms we will translate to their use in electronics. About a decade ago, recycling methods for plastics have been classified into four main techniques: 1) primary, 2) secondary, 3) tertiary, and 4) energy recovery.^[^ ^5^, ^13^ ^]^ García and coworkers, in particular, defined primary recycling as reprocessing to produce a material with the same purpose, while secondary recycling yields a material with different uses than the original plastic.^[^ ^1^ ^]^ The production of plastic bottles made from blends of polyethylene terephthalate (PET) recycled from plastic bottles and virgin PET is an example of primary recycling. Tire recycling is an example of secondary recycling as the vast majority of recycled tires are turned into other rubber products. Both primary and secondary recycling involve mechanical or physical processes, such as grinding, extrusion, and dissolution. However, these processes limit the recyclable materials to mainly thermoplastics, or polymers that can be moldable or reprocessable at elevated temperatures. While primary recycling is referred to as “closed‐loop recycling,” secondary recycling often results in plastics that are lower in quality—that is, polymers with lower molecular weight—and is commonly called “downcycling.” Currently, secondary recycling is the widespread method for large‐scale plastic recycling because achieving identical mechanical properties to those of the original state is limited due to degradation (i.e., chain scissions) or impurities.^[^ ^15^ ^]^ When secondary recycling is not cost‐effective or complicated separations are required, the plastic waste is typically converted into fuel or incinerated.

Tertiary or chemical recycling involves using chemical processes to recover the individual components or monomers. Examples of such processes include hydrolysis, pyrolysis, hydrocracking, and gasification.^[^ ^5^, ^13^, ^16^ ^]^ To expand industrial recycling beyond thermoplastics (primarily PET and polyethylene), ongoing research has focused on catalyst development to improve chemical recycling efficiency and reduce required energy inputs.^[^ ^5^, ^6^ ^]^ While typical tertiary conversion products are liquids and gases, which can be used for feedstock in fuel production, this method has potential for “upcycling” in which case pure monomers are recovered for repolymerization into higher grade products. Whereas downcycling results in polymers with decreased mechanical properties, upcycling can yield polymers with identical or better physical properties. Incineration, also known as quaternary recycling, is a method of energy recovery in the form of heat. While the energy generated from incineration is substantially less compared to the energy conserved from other recycling processes, incineration remains a popular method for waste volume reduction when dealing with mixed and heavily contaminated materials for recycling. Additionally, due to the inevitable release of toxins and greenhouse gases associated with quaternary recycling, it is crucial to develop primary, secondary, and tertiary recycling methods in the advancement of sustainability and energy conservation. To focus on the chemistries involved in furthering recycling efficiency, quaternary recycling will not be covered in this review.

### 2.2 Biodegradable

Biodegradable is a widely used term to classify a range of polymers and polymer composites that can be broken down into smaller constituent pieces under biologically benign or physiological conditions, whether the processes are chemical or biological. At the molecular level, these materials contain chemical linkages that are cleavable in biologically friendly conditions. Biologically friendly conditions include both in vivo degradation and degradation by the natural environment. Chemistries associated with tertiary recycling can also be classified as biodegradable if the conditions are physiologically relevant. Unlike recycling methods, the monomers do not necessarily have to be isolated and collected for further use. At the macroscopic level, biodegradable materials may partially degrade or fully degrade to monomeric units, as defined by Bao and coworkers as type I and type II, respectively.^[^ ^8^ ^]^ Partially degradable (type I) materials are composed of polymers that can disintegrate without full chemical breakdown. In electronics, these materials are typically composed of degradable insulating mediums that hold together nondegradable active materials (i.e., carbon nanotubes, conjugated polymers). For many applications (e.g., implantable electronics, drug delivery), complete degradation into monomeric building blocks may be unnecessary if the polymers break down into substituents that can be either metabolized or excreted. On the other hand, in fully degradable (type II) materials, the polymer backbone can be degraded into oligomers and monomers, enabling potential upcycling or breakdown by microorganisms in the environment. Both the insulating matrix and active materials are degradable in type II electronics. Type II electronics open avenues for reducing electronic waste, recyclability, and improved biocompatibility, as small molecules are less likely to elicit adverse immune response.

Due to the wide appeal of biodegradable polymers for wearable and implantable biomedical applications, biodegradable materials are often studied along with their biocompatibility.^[^ ^2^, ^7^ ^]^ “Bioresorbable” electronics are a specific class of biodegradable materials that can dissolve away in aqueous environments and generate biologically non‐toxic degradation byproducts. Bioresorbable materials can either be type I or type II biodegradable materials, with studies primarily focusing on their dissolution mechanisms.^[^ ^10^, ^11^ ^]^ Thus, biodegradable electronics that directly contact the skin or living tissue require examination of the biocompatibility of the device, degradation intermediates, and polymer byproducts.

### 2.3 Biocompatible

The biocompatibility of electronics must be defined in the context of its location, time of use, and intended application. A material may be biocompatible in one circumstance but not in another. By the IUPAC definition, biocompatibility is the “ability to be in contact with a living system without producing an adverse effect.”^[^ ^14^ ^]^ Generally, a material may be considered biocompatible if it produces an acceptable host response when exposed to the body or bodily fluids. Note that this is distinct from not causing any side effects or immune response. Frequently, biocompatible materials will result in varying degrees of inflammatory and immune responses; however, they are either not harmful or part of the body's normal responses. Signs of adverse response include chronic inflammation, production of cytotoxic substances, cell disruption, skin irritation, restenosis, thrombosis, and corrosion of the implanted material, particularly in the time frame of use and interference with the device function.^[^ ^17^ ^]^ These responses are triggered by chemical or physical reactions to the material. Not only does the material have to be chemically compatible (i.e., hydrophilic, non‐fouling, non‐toxic) with its surrounding environment but also mechanically compatible (i.e., flexible, stretchable, conformal) when considering wearable and implantable electronics. Vigorous assessment by in vitro culture experiments or in vivo implantation is crucial before classifying a material as biocompatible.

## 3 Characterization and Evaluation Methods

The methods of characterization for sustainable polymers encompass a wide variety of techniques and are not standardized partly because there are different targeted environments for each polymer application. For example, standards for biodegradation in marine environments are expected to be different from those in the human body. While systematic metrics are yet to be established, instrumentation are key tools to establish degradation kinetics and identify byproducts. This section first reviews current standards and regulations established by international organizations and federal agencies for commercial plastics and electronic devices. Building off of these policies, we describe relevant experimental characterization methods for new and emerging recyclable, biodegradable, and biocompatible polymers. **Table** **1** summarizes key techniques and instrumentation commonly used to characterize polymeric materials and electronics with elements of recyclability, biodegradability, or biocompatibility.

**Table 1:** Summary of key characterization techniques commonly used to determine recyclability, biodegradability, and biocompatibility

Characterization technique	Information obtained	Recyclability	Biodegradability	Biocompatibility
Dynamic mechanical (thermal) analysis (DMA/DMTA)	Determination of the complex modulus through application of a sinusoidal stress and measurement of strain	✓	—	—
Gel permeation chromatography (GPC)	Determination of molecular weight and dispersity of polymers	✓	✓	—
Nuclear magnetic resonance (NMR)	Identification of small organic compounds through magnetic fields	✓	✓	—
Fourier‐transform infrared spectroscopy (FT‐IR)	Detection of vibration characteristics of chemical functional groups	✓	✓	—
Mass loss profile	Analysis of mass loss via an analytical balance over a specified period	—	✓	—
UV–vis spectroscopy	Analysis of the absorption or reflectance in the UV and visible ranges in which molecules undergo electronic transitions	—	✓	—
Scanning electron microscope (SEM)	Observation of surface topographical changes and composition by scanning with a focused beam of electrons	✓	✓	✓
Light/confocal/difference interference contrast (DIC) microscopy	Imaging of samples through the use of visible light, with increased optical resolution or contrast	—	—	✓
LIVE/DEAD cell viability assay	Determination of cell viability through fluorescent dyes to yield two‐color discrimination of live and dead cells	—	—	✓
Enzyme‐linked immunosorbent assay (ELISA)	Detection of the presence of a protein in a liquid sample using antibodies directed against the protein	—	—	✓
3‐(4,5‐dimethylthiazol‐2‐yl)‐2,5‐diphenyltetrazolium bromide (MTT) assay	Colorimetric assessment of cell metabolic activity via enzymatic reduction of MTT dye to reflect the number of viable cells present	—	—	✓

### 3.1 Characterization and Evaluation for Recyclable Polymers

#### 3.1.1 Current Standards and Regulations

International Organization for Standardization (ISO) 15270 has been established to assist in the development of a sustainable global infrastructure for plastics recovery and recycling.^[^ ^18^ ^]^ The ISO prioritizes the general reduction and optimization of material and energy resource use. The criteria for acceptance of a recycled material, although dependent on the application, may include proper identification, nature and concentrate of contaminants, as well as mechanical and chemical properties and packaging requirements. The recyclate can be used as long as it “meets or exceeds the specified minimum material and end‐use performance criteria.” The Association of Plastic Recyclers (APR) represents companies who acquire, reprocess, and sell more than 90% of the post‐consumer plastic processing capacity in North America. Their Design Guide for Plastics Recyclability considers an item recyclable when at least 60% of consumers have access to a collection system and an item can be further processed cost‐effectively into a post‐consumer plastic feedstock suitable for use in new products.^[^ ^19^ ^]^ Although general guidelines for plastics recycling exist, current policies limit the polymers used to those that are commercially available, such as the well‐known Resin Identification Coding (RIC) system that categorizes plastics into seven groups. The Federal Resource Conservation and Recovery Act of 1976 is the principal federal law in the United States governing the disposal of solid waste and hazardous waste; however, it is difficult to say where polymer‐based electronics fit in as it only covers cathode ray tubes. The majority of states in the United States use the Producer Responsibility approach to hold manufacturers accountable for recycling of their products; however, state electronics recycling policies vary. As the next generation of recyclable plastics and electronics become commercially viable and widespread such that they are a significant waste stream, there must be guidelines for their disposal. We begin to address guidelines for these emerging recyclable materials through the experimental techniques used to characterize them.

#### 3.1.2 Experimental Characterization Methods

**Figure 2:**
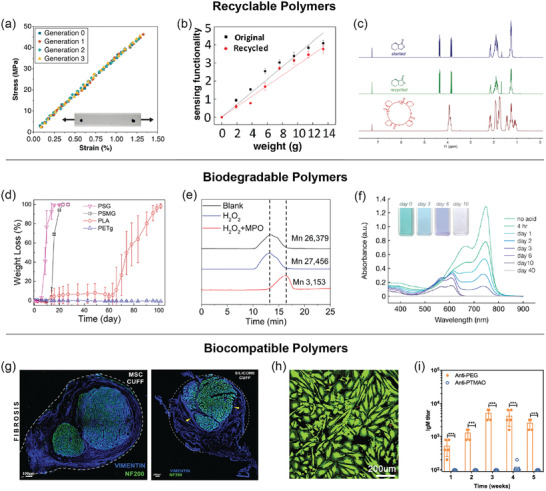
Typical characterization methods used for recyclable, biodegradable, and biocompatible polymers. a) Indistinguishable stress–strain curves of cyclic poly(phthalaldehyde) (cPPA) under quasi‐static tensile loading over three generations of recycling. Adapted with permission.^[^ ^22^ ^]^ Copyright 2019, American Chemical Society. b) Similar sensing performance of the polyimine‐based tactile sensor before and after recycling. Adapted with permission.^[^ ^23^ ^]^ Copyright 2018, AAAS. c) Overlays of ^1^H NMR spectra of starting (blue) and recycled (green) lactone‐based monomers and polymer (red), which display identical chemical shifts (ppm) for the starting and recycled monomers. Adapted with permission.^[^ ^25^ ^]^ Copyright 2018, AAAS. d) Weight loss profiles of polyesters PSG and PSG show significant degradation compared to controls PLA and PETg at 50 °C in phosphate‐buffed saline (pH = 7.4). Adapted with permission.^[^ ^38^ ^]^ Copyright 2020, American Chemical Society. e) GPC traces of the original and degraded SPNs show a reduction of molecular weight based on retention time after treatment with both H_2_O_2_ and myeloperoxidase. Adapted with permission.^[^ ^20^ ^]^ Copyright 2017, Springer Nature. f) UV–vis absorption spectra of a semiconducting imine‐based polymer solution under acidic conditions decreased over 40 d, demonstrating a loss of conjugation. Adapted with permission.^[^ ^4^ ^]^ Copyright 2019, American Chemical Society. g) DIC microscopy images of nerve cross sections with shape memory polymer‐based MSC showed normal nerve fibers (green), while that with a silicone cuff showed nerve compression by fibrotic tissue ingrowth (arrowheads). Dotted lines indicate the relative positions of the MSC and silicone devices. Adapted with permission.^[^ ^49^ ^]^ Copyright 2018, Springer Nature. h) LIVE/DEAD images of cells cultured on PAA‐rGO hydrogel show flourishing cell growth (green) and the absence of cell death (red). Adapted with permission.^[^ ^51^ ^]^ Copyright 2018, Elsevier. i) The titer of PEG‐ and PTMAO‐specific immunoglobin M (IgM) in mice sera was detected with ELISA tests, demonstrating minimal immunogenicity of PTMAO. Adapted with permission.^[^ ^52^ ^]^ Copyright 2019, AAAS.

The specific characterization methods used to determine whether a polymer is recyclable depends heavily on the recycling process used. Common characterization tools for most types of recycling (mechanical and chemical) include gel permeation chromatography (GPC) to compare the initial and recycled polymer molecular weight distributions and polydispersities,^[^ ^20^ ^]^ dynamic mechanical analysis (DMA) for studying the modulus of the material,^[^ ^21^, ^22^ ^]^ and scanning electron microscopy (SEM) to visually observe surface topographical changes.^[^ ^22^ ^]^ When mechanical recycling techniques are employed, mechanical properties and applications of the recycled polymer are compared to the virgin material. For example, a thermally reprocessable rubber crosslinked by dynamic covalent Diels‐Alder bonds was proved dynamically recyclable through their reversible crosslinks which break at 150 °C and re‐form at 50–70 °C. Fourier‐transform infrared (FT‐IR) spectroscopy as well as its mechanical behavior (i.e., tensile testing, hardness and compression set, temperature response) after recycling confirmed reversibility of the network without a reduction in mechanical properties.^[^ ^21^ ^]^ While the un‐crosslinked polymer was soluble in decalin at room temperature, the crosslinked material was not. By dynamic mechanical thermal analysis (DMTA), temperature cycles from 20 to 150 °C of the thermoreversible polymers were performed, showing recovery of the modulus to their original value (93 ± 10%). A study by Moore and coworkers demonstrated the thermal depolymerization and recyclability of self‐immolative, cyclic poly(phthalaldehyde) (cPPA) and its carbon nanofiber‐reinforced composites.^[^ ^22^ ^]^ The depolymerization was monitored by Raman spectroscopy as well as thermal gravimetric analysis (TGA) along with real‐time mass spectroscopy corresponding to the mass loss observed in TGA. The mechanical properties of virgin and recycled materials were evaluated by DMA and quasi‐static tension, which displayed indistinguishable stress‐strain curves over three generations of recycling (**Figure** **2**a). Qualitative morphology evaluation of the carbon fibers after matrix depolymerization was conducted by SEM, in which the fibers were reclaimed and nearly free of residue matrix. For recyclable electronics, such as an electronic skin composed of a dynamic covalent polyimine thermoset,^[^ ^23^ ^]^ characterization of both mechanical properties and functionality (e.g., stress‐strain curves, uniaxial tension tests, electrical resistivity measurements, sensing functionality) were conducted. After recycling three times, stress–strain curves and electrical resistivity of the conductive films did not show noticeable change. The recycled tactile sensor indicated similar sensing performance compared with the original tactile sensor, with a slight reduction in sensitivity likely due to the slight increase in Young's modulus associated with additional crosslinking (Figure 2b). Additionally, a physically crosslinked double network hydrogel used to develop flexible strain sensors was recycled by dissolving in water and subsequent freeze/thaw cycles.^[^ ^24^ ^]^ Comparison of the conductivity, tensile strength and strain, and dynamic rheological properties to those of the original hydrogel showed a recovery of >95% of its mechanical properties.

When chemical recycling processes that produce monomers are used, characterization of the resulting monomer is often included to demonstrate purity for use in repolymerization in addition to examination of the recycled polymer's mechanical properties. For example, gravimetric and NMR analyses showed quantitative monomer recovery without impurities after catalytic chemolysis of lactone‐based polymers (Figure 2c).^[^ ^25^ ^]^ In Helms and coworkers’ recycling of plastics by dynamic covalent diketoenamine bonds, polymers were depolymerized with acid, and components were separated to obtain yields for recovered monomers.^[^ ^26^ ^] 1^H NMR of the monomer showed no detectable side products, residual reagents, or additives. DMA of the recycled material displayed nearly identical properties to the original material. Similarly, García and coworkers compared ^1^H NMR spectra as well as DMA values of their starting and recovered materials to quantitate recyclability.^[^ ^27^ ^] 1^H NMR spectra of the recovered monomer were identical to that of the starting material, while the extracted complex modulus values from stress relaxation after recycling offered comparable performance to the original organogel. Although there is not a standardized method for characterizing recyclable polymers and polymer‐based electronics, these works show that the comparison of mechanical properties and applications of the recycled material after cycling to those of the virgin material constitute enough evidence for recycling. Studies also demonstrate monomer purity if depolymerized monomers can be recovered and separated.

### 3.2 Characterization and Evaluation of Biodegradable Polymers

#### 3.2.1 Current Standards and Regulations

Regulations on the term “biodegradable” have been established to ensure the reliability of products used in the environment. The current American Society for Testing and Materials (ASTM) International policies are defined as standard specifications and test methods. These specifications create a pass or fail situation, while the test methods identify the specific testing parameters such as time frames and toxicity. Under anaerobic conditions, ASTM D5511‐18 and ASTM D5526‐18 indicate that a minimum of 70% of the material should be biodegraded by 30 days (digestion conditions) or the duration of the testing procedure (accelerated landfill conditions).^[^ ^28^, ^29^ ^]^ It is important to note that while the time scales are well defined for in vivo degradation of implantable or digestible materials, the time scales of environmental degradation procedures are more complex. For aerobic environments, ASTM D6400‐19 and ASTM D6868‐19 outline procedures for testing in composting conditions and classify plastics as biodegradable when 90% of the material is fully mineralized into CO_2_ within 180 days.^[^ ^30^, ^31^ ^]^ European and international standards EN 13432:2000 and ISO 14855, respectively, are similar to the described US standards and also determine biodegradability of plastics by analysis of evolved CO_2_.^[^ ^32^, ^33^ ^]^ These standards apply to the more restrictive definition of biodegradability in that the decomposition products are H_2_O, CO_2_, and biomass. Additionally, the standards focus on the resulting products after degradation and not the conditions for testing degradation. As the field of biodegradable polymers for human health applications has only recently emerged, there is a lack of clear procedures to follow for determining if materials are promising for use as implantable or bioresorbable electronics. Potentially, a synergistic fusion of current biodegradable and biocompatible regulations will be a good starting point for this rapidly growing field.

#### 3.2.2 Experimental Characterization Methods

The methods used to characterize biodegradable polymers are similar to those used for chemically recyclable polymers that depolymerize to produce monomers and oligomers, such as TGA and NMR spectroscopy. Biodegradable polymers that are reported without analysis of degradation products are commonly evaluated by mass loss profiles, GPC, UV–vis spectroscopy, SEM, and FT‐IR spectroscopy.^[^ ^3^, ^4^, ^34^, ^35^, ^36^, ^37^ ^]^ Unlike recyclable polymers, characterization for biodegradable polymers does not often include comparison of mechanical properties and functionalities to that of the original material. As biodegradation typically produces monomers or oligomer products, these are commonly analyzed by NMR spectroscopy. However, since the classification of polymers as biodegradable does not require the isolation of the degradation byproducts, separation or extraction processes must also be considered in this case. Instead, biodegradable polymers are often characterized by monitoring the properties of the starting material over time after exposure to a stimulus. Hillmyer, Ellison, and coworkers showed characterization by mass loss of their biorenewable aromatic polyesters poly(salicylic glycolide) (PSG) and poly(salicylic methyl glycolide) (PSMG) after hydrolytic degradation at 50 °C in phosphate‐buffered saline (PBS) solution, artificial seawater, and DI water.^[^ ^38^ ^]^ PSG and PSMG showed significant weight loss at faster time scales compared to polylactic acid (PLA), a representative degradable polyester, and poly(ethylene terephthalate‐*co*‐isophthalate) (PETg), which has similar chemical and physical characteristics to PSG and PSMG (Figure 2d). The supernatants of the degradation solutions were also analyzed by ^1^H NMR to determine that the products were the starting monomers (salicylic acid, glycolic acid, and lactic acid).

Another example of biodegradable characterization was demonstrated by GPC of semiconducting polymer nanoparticles (SPNs), which were used for ultrasensitive in vivo imaging as they eliminate tissue autofluorescence.^[^ ^20^ ^]^ After enzymatic degradation using H_2_O_2_ and myeloperoxidase, GPC traces of the nanoparticles showed a reduction of molecular weight from *M*_n_ ≈ 27–3 kDa (Figure 2e). UV–vis spectroscopy also displayed a decrease in absorption peaks, corresponding to a loss of conjugation associated with depolymerization; however, it is important to note that typically only conjugated polymers have a significant difference between polymeric and monomeric/oligomeric absorption to be analyzed effectively by UV–vis. UV–vis spectroscopy was also used to monitor the degradation of a semiconducting polymer consisting of acid‐labile imine bonds.^[^ ^4^ ^]^ A solution of polymer in 1% 1 m trifluoroacetic acid (TFA) in chlorobenzene showed absorbance reduction and a visual solution color change from blue‐green to purple to clear, corresponding to degradation into monomeric units and eventually ring‐opening of the monomers (Figure 2f).^[^ ^3^, ^4^ ^]^ Additionally, Wallace and coworkers used FT‐IR and SEM to investigate the structural changes after enzymatic degradation of batteries composed of silk fibroin‐polypyrrole films.^[^ ^37^ ^]^ FT‐IR displayed a reduction in the characteristic absorbance band (amide C—N stretching at 1236 cm^–1^) for the amorphous structure of silk, and SEM showed surface erosion of the surface morphology of the film, confirming that the enzyme could penetrate and diffuse inside the swollen film matrix.

Some polymers also allow for characterization techniques that are unique to the polymers’ chemical and physical properties. To probe the enzymatic hydrolysis of polyester elastomers, Sander, Coates, Hillmyer, and coworkers used pH‐stat titration to quantify the number of carboxylic acids formed during polyester hydrolysis and batch reaction vessels coupled to solution total organic carbon (TOC) analysis to quantify soluble hydrolysis products.^[^ ^39^ ^]^ The plateaus of the hydrolysis curves as measured by pH‐stat titration suggested approximately 100% of esters hydrolyzed, at which analysis of the hydrolysis products by ^1^H and ^13^C NMR spectroscopy was consistent with the products expected from full hydrolysis. For solution TOC analysis, the lack of any appreciable dissolved organic carbon after one week of incubation without the enzyme indicated that both abiotic hydrolysis and leaching of organic compounds from the elastomers were negligible. As polymer macroscopic properties are dependent on the molecular and microscopic structure, a loss of functionality also indicates a structural change has occurred. For example, since the SPN‐based afterglow agents previously mentioned exhibit fluorescence and afterglow luminescence, quantification of the radiance intensities showed a decrease after enzymatic degradation via treatment with H_2_O_2_ and myeloperoxidase.^[^ ^20^ ^]^ When characterizing biodegradable electronics, device failure is often additionally noted through manifestation of extreme loss in electronic performance or major substrate damage. For example, degradable cyclic poly(phthalaldehyde) (cPPA) substrates deformed after exposure to UV, causing the resistor to degrade and then fail, which was observed as a sharp increase in resistance.^[^ ^40^ ^]^ In a similar manner, our group designed TFTs based on a biodegradable polymeric substrate and dielectric that irreversibly lost device functionality within two days as the semiconductor delaminated from the dielectric.^[^ ^34^ ^]^ The substrate and dielectric components, however, did not fully degrade until after 30 days. As demonstrated, electronics typically fail before full degradation of the material. Consequently, a loss of functionality alone is not enough to constitute biodegradation of device components.

Due to experimental ease and challenges with isolation and analysis of degradation products, several reported biodegradable polymers only employ mass loss profiles along with some description of change in physical appearance to indicate decomposition.^[^ ^41^, ^42^, ^43^ ^]^ Isolation procedures of the resulting material after degradation treatments alone could produce a mass loss. Batch‐to‐batch variability and the lack of reproducibility associated with simply reporting weight loss necessitate more required standards and methods for the characterization of biodegradable materials. A combination of molecular (i.e., NMR, FT‐IR, GPC), microstructural (i.e., SEM, AFM), and macroscopic (i.e., UV–vis, loss of electronic performance) characterization tools should be employed. Furthermore, for implantable electronics, the toxicity of the degradation products must be examined, much like those used in biocompatibility characterization. Although the biocompatibility of biodegradable electronics is commonly explored in relation to the bulk device before degradation, byproduct toxicity and in vivo studies are crucial in translating these technologies to real‐life applications.

### 3.3 Characterization for Biocompatible Polymers

#### 3.3.1 Current Standards and Regulations

The commercial use of any material in the medical field must meet stringent safety requirements. In terms of regulation of polymeric materials and electronics, the ISO presents widely adopted medical device standards which are addressed in a guidance document called ISO 10993: Biological Evaluation of Medical Devices.^[^ ^17^, ^44^ ^]^ As devices are typically composed of more than one material, it is not sufficient to address the biocompatibility of a single material in relation to a specific location. However, if the material has a proven safe history of medical use, the material characterization phase of evaluation can be omitted.^[^ ^17^ ^]^ These guidelines are currently divided into twenty parts, from animal welfare requirements to principles and methods for immunotoxicology testing of medical devices. The selected test program depends heavily on the material used, contact regime, and time duration of contact with the device. For example, the contact time is broken into short durations (<24 h), prolonged contact (24 h to 30 days), and permanent contact (>30 days). Additionally, biocompatibility protocols must account for potential misuse of the device or material. ISO 10993 is intended to assist developers and manufacturers in designing appropriate testing programs for their engineered device. Other country‐specific guidelines largely overlap with ISO 10993; however, only a couple political regions will be addressed here.

The United States Food and Drug Administration (FDA) uses the United States Pharmacopoeia (USP) 88 Biological Reactivity Tests for in vivo testing.^[^ ^45^ ^]^ Similarly, these tests are directly related to the intended use and location of the plastic component, with different tests for intravenous injection, subcutaneous injection, and implantation. Biocompatibility assessment is conducted through chemical, mechanical, and thermal testing but also includes the effect of (repeated) sterilization procedures on the device. USP 88 categorizes plastics as Class I to VI as well as measures the biological response of animals using standardized temperatures and time regimes. It is important to note that although Class VI plastics must pass the most rigorous testing, it does not fully meet any category of ISO 10993 testing guidelines. European Union device manufacturers are currently governed by Regulations (EU) 2017/745 and 2017/746 for general medical devices and in vitro diagnostic medical devices, respectively.^[^ ^46^, ^47^ ^]^ These regulations provide clearer requirements for clinical data on medical devices and their assessment as well as enhanced provisions for post‐market surveillance. Collectively, these organizations address nearly all conceivable medical device testing concerns.

#### 3.3.2 Experimental Characterization Methods

Since the immunological responses of the human body are complex, biocompatibility must be considered in relation to many different cell types and sites of application. Although the biocompatibility of electronics used in the biomedical field is highly dependent on the application of the device, biocompatible electronics developed in research mainly use a combination of microscopy/imaging techniques (e.g., brightfield/light, fluorescence, SEM, confocal) as well as various cell assays to test for viability. Examples of imaging techniques used for polymeric materials are described herein. In a study of rat hippocampal neurons cultured on flexible 3D pillar electrodes made of polydimethylsiloxane (PDMS), brightfield and confocal fluorescent microscopy images were taken.^[^ ^48^ ^]^ Visual assessment confirmed healthy neuron growth with typical morphology, including formation of a confluent monolayer of cells and extensive branching neurites. Through a different imaging technique, thin shape memory polymer‐based multi‐electrode softening cuffs (MSCs) were compared to silicone analogues for fibrotic growth after implantation on somatic nerves.^[^ ^49^ ^]^ Staining of adjacent tissue and comparison of differential interference contrast (DIC) microscopy images showed a reduction in inflammatory cells evoked by the MSC device (Figure 2g). These MSCs were not only chemically biocompatible but also mechanically biocompatible through their conformability. Traeger, Schubert, and coworkers employed high resolution microscopy to examine the gene transfection mechanism of a modified poly(ethylene imine) (PEI) copolymer.^[^ ^50^ ^]^ Confocal microscopy, structured illumination microscopy with fluorescence imaging, and high‐angular annular dark‐field scanning transmission electron microscopy (HAADF‐STEM) of embedded cell samples revealed a co‐localization of DNA‐bound polymers within lysosomes, showing efficient release of DNA into the cytoplasm. In addition to cytotoxicity studies by cell incubation and viability, the blood compatibility of the PEI copolymer was assessed by a hemolysis assay, which determined low hemolytic activity as well as minimal aggregation of erythrocytes.

The in vitro and in vivo tests for biocompatible polymers and electronics vary greatly, especially due to the location in the body the material or device is intended to be used. A polyacrylic acid (PAA)/reduced graphene oxide (rGO) nanocomposite hydrogel used for wearable strain sensors was evaluated for biocompatibility through in vitro HEF1 fibroblast cell cultures.^[^ ^51^ ^]^ Cell viability was investigated using a commercial LIVE/DEAD Viability/Cytotoxicity Kit, which simultaneously displays both live (green) and dead (red) cells by the targeting of different fluorescent dyes. In this kit, the green fluorescent calcein‐AM dye targets esterase activity in the cytoplasm of living cells, while the red fluorescent ethidium homodimer (EthD‐1) dye demonstrates cell death by penetrating damaged cell membranes. After staining, the cells were observed using confocal microscopy, demonstrating over 95% cell viability and flourishing cell growth after 14 days (Figure 2h). Poly(trimethylamine *N*‐oxide) (PTMAO), an ultralow fouling polymer reported by Jiang, was tested for in vitro fouling through incubation with plasma proteins and measurement by enzyme‐linked immunosorbent assay (ELISA).^[^ ^52^ ^]^ Comparison of the developed polymer's protein and cell adsorption to that of commercial polymers displayed PTMAO's exceptional nonfouling capability. The nonfouling property was further tested in undiluted human blood serum, which is known as the most challenging in vitro system as it closely mimics humans’ complex biological environment. Surface plasmon resonance (SPR) binding analysis was used to detect low protein adsorption levels. Subcutaneous and intravenous injections in mice also indicated minimal immunogenicity by antibody tests by ELISA (Figure 2i). For a different PEI copolymer, a 3‐[4,5‐dimenthylthiazol‐2‐yl]‐2,5‐diphenyltetrazolium bromide (MTT) assay using human embryonic kidney (HEK) 293 cells was conducted to investigate in vitro cytocompatibility.^[^ ^53^ ^]^ The interactions of this polymer with serum proteins were assessed by dynamic light scattering (DLS) measurements for particle size instead of SPR analysis, indicating serum stability and the absence of harmful serum coagulation. Although these methods vary widely, many studies similarly use a mix of imaging techniques and cell viability assays to compare the developed biocompatible polymeric material to its non‐biocompatible counterparts. The actual methods picked are specific to the material's intended application either on the skin, in bodily fluids, or elsewhere in the body.

## 4 Molecular Design of Polymers

From the molecular level, the chemical structures of polymers intrinsically determine their microscopic and macroscopic properties. The structure–property relationships of these polymers highlight the molecular design of polymers that exhibit recyclability, biodegradability, or biocompatibility. Typically, molecular design focuses on a bottom–up strategy, where the polymer repeating units are designed, tailored, and further polymerized into either linear or network structures. Understanding the molecular design of polymer structures allows for tuning of the polymer architecture as well as the development of materials with emergent, desired functionalities not already available in commercial polymers. In this section, we introduce basic established chemistries for each of these eco‐ and human‐friendly categories and then examine emerging polymers in each field and their advantages over existing chemistries. These basic chemistries cover commonly used functional groups as well as traditional polymers (i.e., commercial, naturally derived, or discovered over a decade ago), while emerging polymers are synthetically designed as recent, sustainable advances over the past few years.

### 4.1 Recyclable

#### 4.1.1 Chemistries for Traditional Recyclables

**Figure 3:**
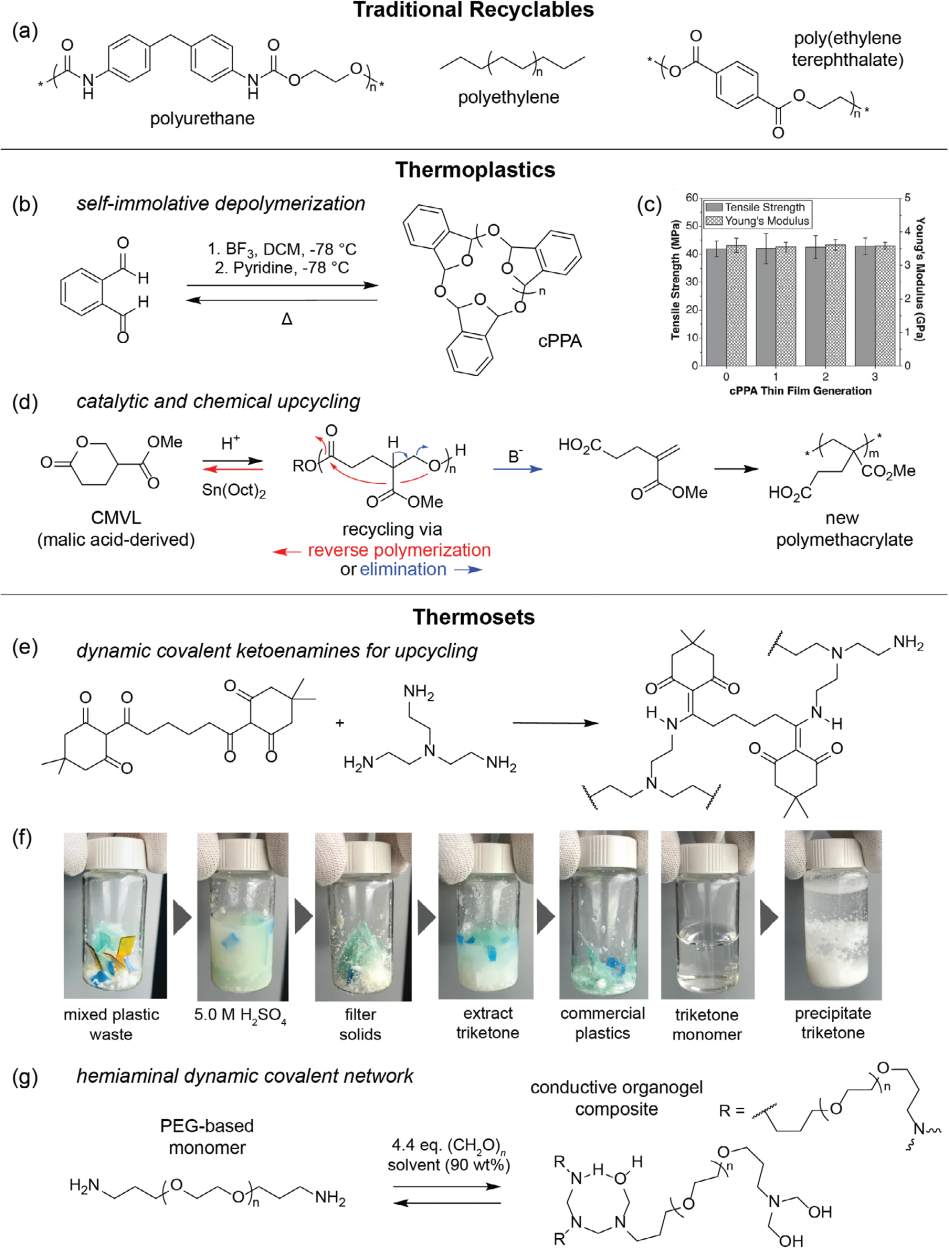
Molecular design for traditional recyclables as well as emerging thermoplastic and thermosetting recyclable polymers. a) Traditional recyclable polymers include poly(ethylene terephthalate), polyethylene, and polyurethane. b) Thermally‐mediated polymerization and depolymerization of self‐immolative cPPA. c) Mechanical properties of the virgin and recycled cPPA thin films. Adapted with permission.^[^ ^22^ ^]^ Copyright 2019, American Chemical Society. d) Divergent chemical recycling of a polyvalerolactone to CMVL monomer or new polymethacrylate. Adapted with permission.^[^ ^64^ ^]^ Copyright 2018, American Chemical Society. e) Scheme of recyclable network PDKs synthesized from polyamines and ditopic triketones. f) Photographs showing orthogonal depolymerization of PDKs and triketone monomer recovery from mixed plastic waste. Adapted with permission.^[^ ^26^ ^]^ Copyright 2019, Springer Nature. g) Scheme of reversible hemiaminal network synthesized from a PEG‐based monomer. Adapted with permission.^[^ ^27^ ^]^ Copyright 2019, Wiley‐VCH.

Traditional recyclables are classified as plastics that are in widespread and commercial use, such as those in the RIC system. Most conventional recyclable linear polymers/thermoplastics (e.g., polyethylene, polyurethane, PET) are often subjected to physical downcycling processes, which unfortunately lead to the deterioration of mechanical properties through discoloration or decreases in molecular weight (**Figure** **3**a). Additionally, thermal and catalytic pyrolysis at high temperatures (>400 °C) for polymers such as polyethylene suffer from low energy efficiency and lack of product control, resulting in complex product compositions that are difficult to separate for future use.^[^ ^54^ ^]^ The high cost and energy consumption associated with downcycling and more common tertiary recycling methods have led to the recent exploration in improving catalytic recycling design. For example, Guan, Huang, and coworkers developed an iridium catalytic system based on alkane cross‐metathesis for the facile recycling of commercial polyethylene into useful liquid fuels and waxes under mild conditions.^[^ ^54^ ^]^ This method also showed excellent degradation product distribution (liquid fuels versus waxes) through control of the catalyst structure and reaction time. Although the above secondary and tertiary recycling strategies have been advanced to improve recycling efficiency, they still typically lead to some unrecyclable impurities or only convert polymers into small‐molecule derivatives that require additional modification to be converted into polymerizable monomers.^[^ ^55^, ^56^ ^]^ One category of chemical recycling that has been gaining attention is the upcycling of polymers through the reclamation of pure monomers. As a specific example, Hedrick and coworkers investigated the organocatalyzed aminolysis of PET, one of the most recycled commercial thermoplastics, to produce a broad range of terephthalamide monomers.^[^ ^57^ ^]^ On the other hand, the recycling of thermosets is challenging as they are typically permanently crosslinked and unlikely to be reprocessable via conventional methods. Hence, the expansion of recyclable thermosets as well as polymers that can be fully recycled into polymerizable monomers are desirable in terms of procedure simplification, energy cost, and the realization of sustainable materials with a broad range of mechanical properties.

#### 4.1.2 Molecular Design of Recyclable Polymers

In discussing the molecular design of recyclable polymers in recent years, we will address various types of recyclable polymers (e.g., self‐immolative, linear, thermosetting) and include one or more examples in each category.

##### Self‐Immolative Polymers

With the goal of a circular plastics economy, the upcycling of polymers into reusable monomers or starting materials has been emerging as an attractive topic. In the last decade, vast attention has been paid to the development of self‐immolative polymers, which are a class of metastable polymers that self‐depolymerize into monomers upon external stimuli (e.g., backbone cleavage or removal of chain‐end capping functionality).^[^ ^58^ ^]^ Moore and coworkers employed phthalide monomers for the preparation of self‐immolative cyclic poly(phthalaldehyde) (cPPA), of which the facile depolymerization and repolymerization was demonstrated for the recycling of carbon fiber‐reinforced cPPA composites.^[^ ^22^ ^]^ cPPA depolymerized through cleavage of the acetal backbone at 120 °C in only 14 min, with simultaneous quantitative monomer recovery (Figure 3b). Both cPPA thin films and the reinforced composites retained >99% of their moduli and tensile strength after multiple recycling steps (Figure 3c). Interestingly, although thermal recycling methods are conventionally considered secondary recycling, self‐immolative polymers form a special class of recyclable polymers that allow for upcycling under broader recycling conditions. Additionally, changing the initiator during polymerization has been demonstrated to produce linear phthalide‐based polymers installed with various end‐caps that allow for triggered self‐degradation in response to different stimuli.^[^ ^59^, ^60^ ^]^ These phthalide‐based polymers possess a low ceiling temperature, owing to the metastable characteristic of their polymer backbone, and thus the working temperature range of the polymer needs to be considered in the design of thermally recyclable materials.

##### Thermoplastics

As the intrinsic instability of self‐immolative polymers limits their practical applications, recyclable polymers with a higher thermal stability are more desirable. To address this issue, Chen and coworkers introduced polylactone‐family polymers based on *γ*‐butyrolactone and its derivatives, in which the catalyst or monomer design allows for facile ring‐opening polymerization (ROP) to yield depolymerizable polylactones.^[^ ^25^, ^61^, ^62^ ^]^ Both linear and cyclic polymers were synthesized, depending on the type of applied catalyst, and the resulting polymers showed enhanced thermostability and repeatable and quantitative recyclability by thermolysis or chemolysis.^[^ ^25^ ^]^ In the presence of a catalytic amount of ZnCl_2_, the temperature required for recycling for both linear and cyclic polymers decreased from ≥300 to 120 °C. Consecutive polymerization‐depolymerization showed quantitative monomer recovery (97%) and reproducible, subsequent monomer conversion (85%) over three cycles. This work contributes to the efforts in achieving circular monomer–polymer–monomer cycles, a challenge in the development of chemically recyclable polymers.

While the synthetic design of new recyclable polymers can often be expensive, the modification of renewable or biobased polymerizable monomers offers further sustainability in addition to upcycling. Notably, commercial polyurethanes are commonly used in coatings, adhesives, sealants, elastomers, and foams; however, their resistance to degradation results in significant environmental challenges with millions of tons produced annually. Hillmyer and coworkers developed chemically recyclable thermoplastic polyurethanes (TPUs) and flexible foams from a depolymerizable polyester poly(*β*‐methyl‐*δ*‐valerolactone) (PMVL).^[^ ^63^ ^]^ PMVL monomers were easily synthesized from sugar and estimated to be low in cost (≈$2 kg^−1^). The formed TPUs were linear with urethane‐rich segments, with high toughness and elasticity, mimicking commercial TPUs. In contrast, the PMVL foams were composed of branched networks with hard and soft segments. Due to the reversibility of urethane bonds, thermodynamic depolymerization of these foams occurred by heating at 200–250 °C at ≈100 mTorr, and the presence of catalytic Sn(Oct)_2_ facilitated depolymerization rates. This recycling method does not require the addition of any solvents, and pure monomers can be regenerated by distillation without a loss in purity. More recently, Hoye and coworkers prepared another substituted polyvalerolactone made from 4‐carbomethoxyvalerolactone (CMVL), a renewable monomer synthesized from malic acid in two steps.^[^ ^64^ ^]^ This CMVL polymer goes through divergent chemical recycling through two independent pathways (Figure 3d). The first similarly uses Sn(Oct)_2_ with heating (150 °C) for a backbiting depolymerization to form the original monomer, while the second uses base to cleave the polyester through a retro‐oxa‐Michael reaction, producing a methacrylate analogue that could readily undergo radical polymerization to give a new polymethacrylate. The demonstrated catalytic strategies and divergent recycling realizes sustainable, high‐performance polymers designed to fit a circular economy and can be further extended to other polyols to create a wide range of recyclable materials.

##### Thermosets

The recycling of thermosets/polymer networks is usually more challenging as these polymer networks are typically permanently crosslinked, and therefore, more stable and resistant compared with thermoplastics. The introduction of dynamic covalent chemistries into polymer networks allows these thermosetting materials to be thermally processed and recycled like thermoplastics. At ambient temperatures, these reversibly crosslinked polymers behave as typical strong thermosets due to the favored association under equilibrium or slow exchanging dynamics. In contrast, with fast exchanging or enhanced dissociation at elevated temperatures, these dynamic networks can flow quickly and thus be reprocessed. The Diels–Alder reaction, a well‐documented dynamic chemistry, has been widely applied as a thermoreversible crosslinking tool owing to its easy chemistry, fast kinetics, and mild reaction conditions.^[^ ^21^, ^65^ ^]^ Wudl's seminal work on fully reversible, self‐healable Diels–Alder crosslinked networks,^[^ ^66^ ^]^ together with Liebler's later work using dynamic transesterification,^[^ ^67^ ^]^ paved the way toward new crosslinking strategies for a variety of recyclable rubber products. Picchioni and coworkers functionalized a commercial ethylene‐propylene rubber with furan groups, which were then crosslinked with bismaleimide through Diels–Alder cycloaddition.^[^ ^21^ ^]^ The resulting crosslinkers dissociated at elevated temperatures (>150 °C) after one hour, and the subsequent thermal annealing allowed for re‐formation of networks. This dissociation‐reformation feature of the polymer network allows the material to be recycled using hot‐press, a secondary recycling method. Specifically, the material was cut into pieces and subjected to subsequent compression molding to yield new samples with comparable mechanical properties—a feat impossible with conventional synthetic rubbers. Other reversible chemistries, including transamination,^[^ ^68^ ^]^ disulfide exchange,^[^ ^69^ ^]^ siloxane exchange,^[^ ^70^ ^]^ and dioxaboralane metathesis^[^ ^71^ ^]^ have also been explored in producing self‐healing or recyclable polymer networks.

While existing strategies to create reprocessable thermosets have focused on dynamic covalent bond exchange and thermomechanical degradation, Johnson and coworkers provided a complementary approach using mild, chemically triggered network degradation, and the degradation products were recycled as valuable starting materials.^[^ ^72^ ^]^ The authors selectively installed a small quantity of cleavable bonds within the backbone of an industrial thermoset polydicyclopentadiene (pDCPD) to yield recyclable products of controlled molecular weight and functionality. Altering the loading of cleavable silyl ether monomers for copolymerization with norbornene derivatives by ring‐opening metathesis polymerization (ROMP) produced degradable statistical copolymers. Excess tetrabutylammonium fluoride selectively cleaved these copolymers in 4 h at a low 10% cleavable monomer loading. For comparison, the authors explored introducing silyl ethers as crosslinkers between polynorbornene strands, which did not degrade even at 80% cleavable crosslinker loading. They recycled and copolymerized the degradation products with fresh DCPD monomers to yield new materials with comparable stress–strain behavior and elastic moduli relative to those of the virgin material. Thermoset composite recycling through the introduction of cleavable bonds within the polymer backbone can impart degradability and recyclability at low co‐monomer loadings, whereas the analogous addition of cleavable crosslinks cannot.

Additionally, while most reported dynamic covalent networks addressed their reprocessability, less attention has been paid to the recovery of the network into reusable building blocks or monomers. Another example of chemical degradation of thermosetting polymers by Helms and coworkers described recyclable networks based on dynamic covalent diketoenamine bonds, which undergo reversible depolymerization to give high‐value monomers in the recycling process.^[^ ^26^ ^]^ In detail, *β*‐triketones and aromatic/aliphatic amines underwent a facile “click” reaction through ball‐milling to form poly(diketoenamine)s (PDKs), producing water as the only byproduct (Figure 3e). These PDKs were hydrolyzed in strong aqueous acid (0.5–5.0 m H_2_SO_4_) at ambient temperature and further treated to regenerate triketone and amine monomers. To demonstrate the facile recycling of these PDKs, they physically mixed these PDKs with commercial plastics, which do not depolymerize under the same recycling conditions, and treated the mixture with recycling procedures (Figure 3f). Desirably, PDKs were selectively dissembled from the mixed plastic waste streams, and monomers were cleanly retrieved. This selectivity in recycling highlights the simplicity of raw‐material recovery from plastic waste mixtures, of which the purification or recovery procedures are typically tedious, complex, and highly energy demanding. The facile, clean, and selective recycling features “closed‐loop recycling” of thermosetting materials and renders these plastics as promising polymers with minimal environmental impact. While the authors refer to this process as closed‐loop recycling, this term was previously only used to refer to primary recycling, which involves physical or mechanical processes. Perhaps with the rapid advancement of chemical recycling, the term is evolving to encapsulate all polymers capable of regenerating the original material without a loss in quality regardless of the process used.

Recyclable thermosets that are also conductive are even rarer. Building from previous work on recyclable thermosetting polymers,^[^ ^73^ ^]^ García and coworkers further demonstrated organogel composites that are both conductive and fully recyclable.^[^ ^27^ ^]^ They found that polymer composites comprised of a hemiaminal dynamic covalent network (HDCN) and fillers could exhibit high conductivities up to 9.95 mS cm^–1^, which are suitable for sensing applications. Various conductive fillers (e.g., carbon nanotubes, carbon black, graphite) and solvents were screened for in the synthesis of HDCNs based on polyethylene glycol (PEG) monomers (Figure 3g). The resulting conductivity, modulus, and relaxation time can be effectively tuned based on filler selection, which highly impacts the interaction strength with the polymer matrix. While HDCNs synthesized in *N*‐methyl‐2‐pyrrolidone (NMP), *N*‐cyclohexyl‐2‐pyrrolidone, dimethylformamide, and dimethyl sulfoxide were recyclable in pH‐neutral water with 95% monomer recovery, conductive filler‐matrix networks could be recycled using dilute acid (pH = 3) via hydrolysis within 20 min. These organogel composites enable desirable conductive functionalities and potential applications for recyclable thermosets to be used in electronics.

### 4.2 Biodegradable

#### 4.2.1 Basic Chemistries for Biodegradables

**Figure 4:**
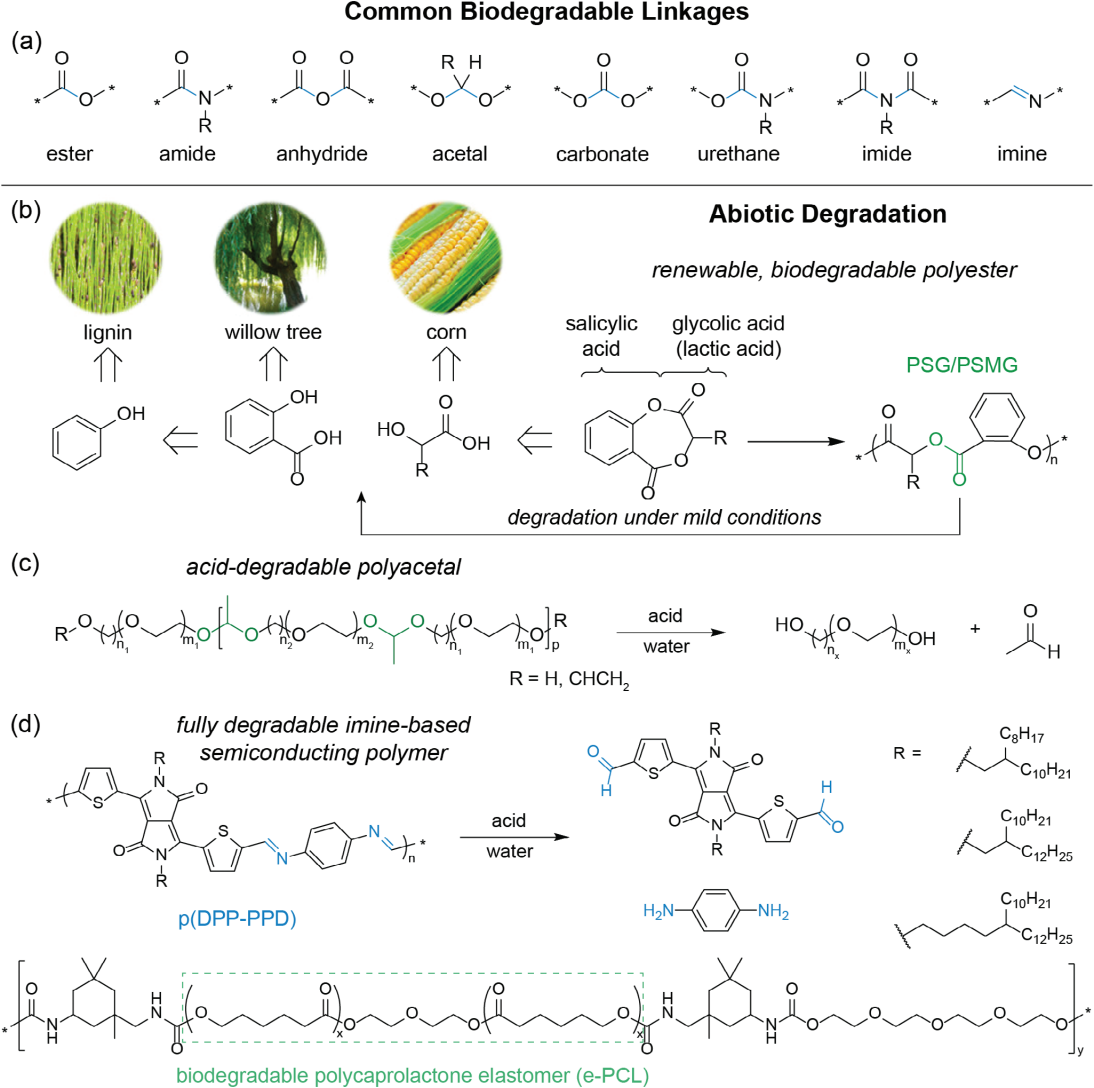
Chemistries of common biodegradable linkages and molecular design for emerging abiotically biodegradable polymers. a) Chemical structures of moieties susceptible to hydrolytic cleavage, which are commonly used in biodegradable polymers. Blue shows the bond(s) being broken. b) Scheme of renewable, biodegradable aromatic polyesters synthesized through ROP of a biobased lactone monomer. Adapted with permission.^[^ ^38^ ^]^ Copyright 2020, American Chemical Society. c) Scheme of water‐soluble, temperature‐responsive polyacetals and their degradation via acid hydrolysis. Adapted with permission.^[^ ^36^ ^]^ Copyright 2016, American Chemical Society. d) Chemical structures of the fully degradable semiconducting polymer p(DPP‐PPD) and its monomeric byproducts after cleavage as well as biodegradable elastomer e‐PCL. Adapted with permission.^[^ ^4^ ^]^ Copyright 2019, American Chemical Society.

Common biodegradable polymers are derived from naturally occurring materials, such as plant‐based cellulose and dextran as well as animal‐derived collagen and silk fibroin. On the other hand, biodegradable synthetic polymers offer more control of the polymer architecture, and thus, degradation kinetics and mechanical properties. Most explored biodegradable polymers are based on hydrolytically cleavable linkages, including ester, amide, anhydride, acetal, carbonate, urethane, imide, and imine bonds (**Figure** **4**a). Polylactic acid (PLA, PDLA, PLLA), poly(lactic‐*co*‐glycolic acid) (PLGA), and poly(caprolactone) (PCL) are well‐established degradable polymers that take advantage of their cleavable ester bonds.^[^ ^6^, ^12^ ^]^ However, the degradation of these polymers in landfills is highly dependent on the molecular weight, temperature, humidity, and oxygen availability and does not occur under common environmental conditions.^[^ ^74^ ^]^ In addition to common polyesters, biodegradable elastomers with flexible and stretchable mechanical properties have also been explored. A widely used biodegradable elastomer in biomedical applications, poly(glycerol sebacate) (PGS), was first introduced by Langer and coworkers in 2002.^[^ ^41^ ^]^ Compared with biodegradable elastomers at the time, the covalent crosslinks in PGS provided both toughness and flexibility, which are ideal for implantable devices that undergo large deformations in the body's dynamic environment. The degradation of PGS occurred through surface decomposition instead of bulk degradation, in which mechanical properties decrease abruptly.^[^ ^75^ ^]^ In vivo degradation of PGS samples in rats were fully absorbed in 60 days. In contrast, agitation for the same amount of time in a PBS (pH = 7.4) solution only resulted in ≈17% degradation by mass loss. There is much room for improvement in the molecular design of biodegradable polymers with more robust and controlled degradation as well as uniformity to ensure a reproducible biological response for implantable applications. While there are also moieties susceptible to oxidative and reductive cleavage,^[^ ^76^, ^77^, ^78^, ^79^ ^]^ redox‐responsive polymers for biodegradation have been less studied. Additionally, emerging synthetic polymers have been designed to degrade when stimulated by light as well as to be enzymatically degradable, some of which will be explored in the next section.

#### 4.2.2 Molecular Design of Biodegradable Polymers

##### Abiotic Degradation

Synthetic biodegradable polymers are typically abiotically degraded, in which chemical and physical conditions not derived from living organisms are used. Among the different classes of synthetic polymers, polyesters are the most studied for biomedical applications. Traditional aromatic polyesters, such as PET, make up close to 10% of the global plastic market, are not readily degradable, and are derived from non‐renewable resources. Over the past few years, tremendous effort has been directed at designing these polyesters to be renewable and biodegradable. Miller and coworkers copolymerized camphoric acid, an inexpensive and biorenewable diacid from camphor laurel trees, with various diols to afford copolymers with a large range of glass transition temperatures (*T*_g_).^[^ ^80^ ^]^ Polyethylene camphorate was degraded by agitation in aqueous solutions of pH = 1 and pH = 2 and deionized (DI) water at pH = 7 for 14 days at room temperature. GPC analysis after DI water treatment showed a reduction in *M*_n_ from 20,200 to <600 Da. In a further attempt to address the feasibility of using camphoric acid to replace the phthalic acid in PET, copolymers comprised of both acid blocks were successfully prepared. However, the increase in biobased camphorate incorporation decreased the *T*_g_ from that of PET from 71 to 41 °C, requiring further tuning of the polymer architecture to serve as an alternative to PET in terms of its physical properties. Recently, Hillmyer, Ellison, and coworkers also synthesized biodegradable polyesters from sustainable feedstocks that rapidly degrade under mild conditions (Figure 4b).^[^ ^38^ ^]^ Aromatic polyesters derived from salicylic acid, poly(salicylic glycolide) (PSG) and poly(salicylic methyl glycolide) (PSMG), were found to have comparable glass transition temperatures and Young's moduli to those of PET. PSG and PSMG were immersed in PBS solution (pH 7.4), artificial seawater (pH = 8.0), DI water (pH = 7.1) and 0.1 m NaOH at 50 °C. Both samples showed significant weight loss within 30 days compared to PLA, which took twice as long to degrade. These biorenewable polyesters have facile degradation properties that could substitute for nondegradable PET derivatives. While it is not necessary for biodegradable polymers to be also biobased, we highlight these select efforts in achieving fully sustainable polyesters.

Polyacetals are another class of polymers that have been synthetically designed to exhibit biodegradability as the ketal functionalities are typically degradable under mildly acidic conditions. Koberstein and coworkers demonstrated a new family of acid‐degradable polyacetals with lower critical solution temperature (LCST) behavior owing to the presence of both hydrophobic and hydrophilic blocks along the polymer chain, and the polyacetals were found to possess predictable temperature response (Figure 4c).^[^ ^36^ ^]^ These water‐soluble, temperature‐responsive polymers have potential applications in tissue scaffolds, actuators/artificial muscles, and drug delivery vehicles. The synthesized polyacetals were relatively stable under neutral conditions, with a molecular weight decrease of ≈10% after 3 days at pH = 7.4. In contrast, the molecular weight decreased by ≈70% after 3 days at pH = 6.5, and the polymer completely degraded after 3 days at pH = 5.5. These mildly acidic conditions are analogous to areas in the human body, such as the upper stomach (pH = 4.0–6.5), endosomes and lysosomes (pH = 4.5–5.5), and tumor tissues (pH = 4.2–6.7).^[^ ^81^ ^]^ Polyesters and polyacetals are typically synthesized through ROMP or step‐growth polymerizations, which often give fairly broad polydispersities of ≈1.5–2. For better control of polydispersities and design of the polymer architecture (e.g., block copolymer, star/branch structure), living chain polymerizations have been applied for the preparation of biodegradable materials. As the biodegradability of polymers has been shown to be dependent on their molecular weight,^[^ ^82^, ^83^ ^]^ polymers with narrow polydispersities can be anticipated to give more controlled biodegradation. Gutekunst and coworkers achieved rapid and living polymerizations of polyacetal materials using modular enyne monomers.^[^ ^35^ ^]^ The obtained degradable polymers possess narrow polydispersities of ≈1.1–1.5 and exhibit optimal degradation in the presence of acetic acid (AcOH) or trifluoroacetic acid (TFA). The polyacetal showed gradual hydrolysis in neutral conditions, with a 63% reduction in molecular weight after 48 h by GPC. The addition of AcOH resulted in a higher 80% mass reduction after 48 h, while TFA caused rapid degradation into small molecules after 24 h. Although a large amount of organic solvent was needed for the polymers to dissolve due to their hydrophobic nature, the enyne monomers can be designed to be more hydrophilic.

The integration of fully biodegradable electronics calls for polymer semiconductors and conductors that are also biodegradable. However, most reported biodegradable polymers are insulating polymers. Designing biodegradable electronically‐active conjugated polymers involve the incorporation of degradable bonds that maintain conjugation into the polymer backbone. Our group incorporated hydrolyzable imine linkages, which preserve conjugation, into the backbone of conjugated donor–acceptor polymers to achieve transient semiconductors. These acid‐labile semiconducting polymers were further blended with a biodegradable elastomer to achieve semiconductors that are both stretchable and fully degradable.^[^ ^4^ ^]^ The molecular design involved a dialdehyde‐functionalized diketopyrrolopyrrole (DPP) and *p*‐phenyldiamine, which were polymerized by imine condensation to form p(DPP‐PPD). Upon spin‐coating a solution of p(DPP‐PPD) and urethane‐based elastomer (e‐PCL), the thin film exhibited self‐assembled nanoconfined fibril aggregates of p(DPP‐PPD) embedded within the e‐PCL matrix (Figure 4d). A solution of neat p(DPP‐PPD) in 1% 1 m TFA in chlorobenzene showed peak maxima in UV–vis absorption spectra diminishing completely after 10 days, with all absorption peaks becoming negligible after 40 days. Thin films of both neat and nanoconfined p(DPP‐PPD) in water with 0.1 m TFA displayed similar trends of peak maxima decreasing after 10 days. This biodegradable active material further advances the development of new multifunctional technologies for human health and environmental sustainability.

##### Biodegradation

**Figure 5:**
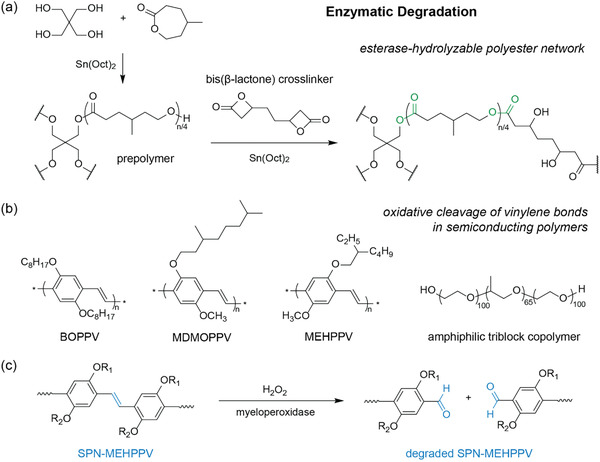
Molecular design for emerging enzymatically biodegradable polymers. a) Synthesis of a renewable polyester network obtained through ROP of derived caprolactone and subsequent crosslinking with a bis(*β*‐lactone) monomer. Adapted with permission.^[^ ^39^ ^]^ Copyright 2018, American Chemical Society. b) Chemical structures of the semiconducting polymer nanoparticles and triblock copolymer. c) Scheme of the degradation of SPNs, which contain vinylene bonds that are cleavable in the presence of oxidative species and myeloperoxidase. Adapted with permission.^[^ ^20^ ^]^ Copyright 2017, Springer Nature.

Unlike the previously discussed abiotic degradation studies, biodegradation in natural systems is inherently biologically benign as it is achieved through microorganisms and their enzymes. Sander, Coates, Hillmyer, and coworkers designed chemically crosslinked polyester elastomers that are renewable and enzymatically hydrolyzable.^[^ ^39^ ^]^ ROP of *γ*‐methyl‐*ε*‐caprolactone generated prepolymers, which were then crosslinked using a novel bis(*β*‐lactone) crosslinker (**Figure** **5**a). The obtained polyester networks were subjected to degradation experiments using *Fusarium solani* cutinase (FsC), an esterase (hydrolase enzyme) from filamentous fungi, and were found to readily hydrolyze at neutral pH and environmentally relevant temperatures (2−40 °C). In contrast, abiotic degradation of traditional polyester networks typically requires much harsher conditions (e.g., strong acid or base, temperatures >200 °C). The extracellular esterases cleaved the polyesters into smaller, water‐soluble monomeric units of hexanoic acids and oligomers that can be taken up and used by microorganisms, producing CO_2_ and microbial biomass.^[^ ^84^ ^]^ Complete degradation was achieved at temperature‐dependent rates, with higher temperatures giving faster hydrolysis rates.

Enzymes are also capable of chemical reactions at physiological conditions that would otherwise require harsh or non‐biocompatible conditions abiotically. Pu and coworkers took advantage of enzymatically biodegradable vinylene bonds that undergo oxidative cleavage by H_2_O_2_ and myeloperoxidase.^[^ ^20^ ^]^ The authors designed semiconducting polymer nanoparticles (SPNs) that store photon energy and emit long‐NIR afterglow luminescence for applications in ultrasensitive in vivo optical imaging. These phenylenevinylene‐based SPNs (BOPPV, MDMOPPV, MEHPPV) were transformed into water‐soluble nanoparticles in the presence of an amphiphilic triblock copolymer (Figure 5b). They treated the SPN solutions to H_2_O_2_ and myeloperoxidase at 37 °C for 8 h in PBS solution (Figure 5c). GPC, UV–vis spectroscopy, and quantification of the fluorescence and afterglow luminescence intensities of the original SPNs, SPNs treated with H_2_O_2_, and SPNs treated with both H_2_O_2_ and myeloperoxidase were used to analyze successful degradation of the polymer. As H_2_O_2_ is produced naturally in the lungs, gut, and thyroid gland of humans, these semiconducting polymers have potential to be used for implantable electronics in certain locations in the body.

### 4.3 Biocompatible Polymers

#### 4.3.1 Basic Chemistries for Biocompatible Materials

**Figure 6:**
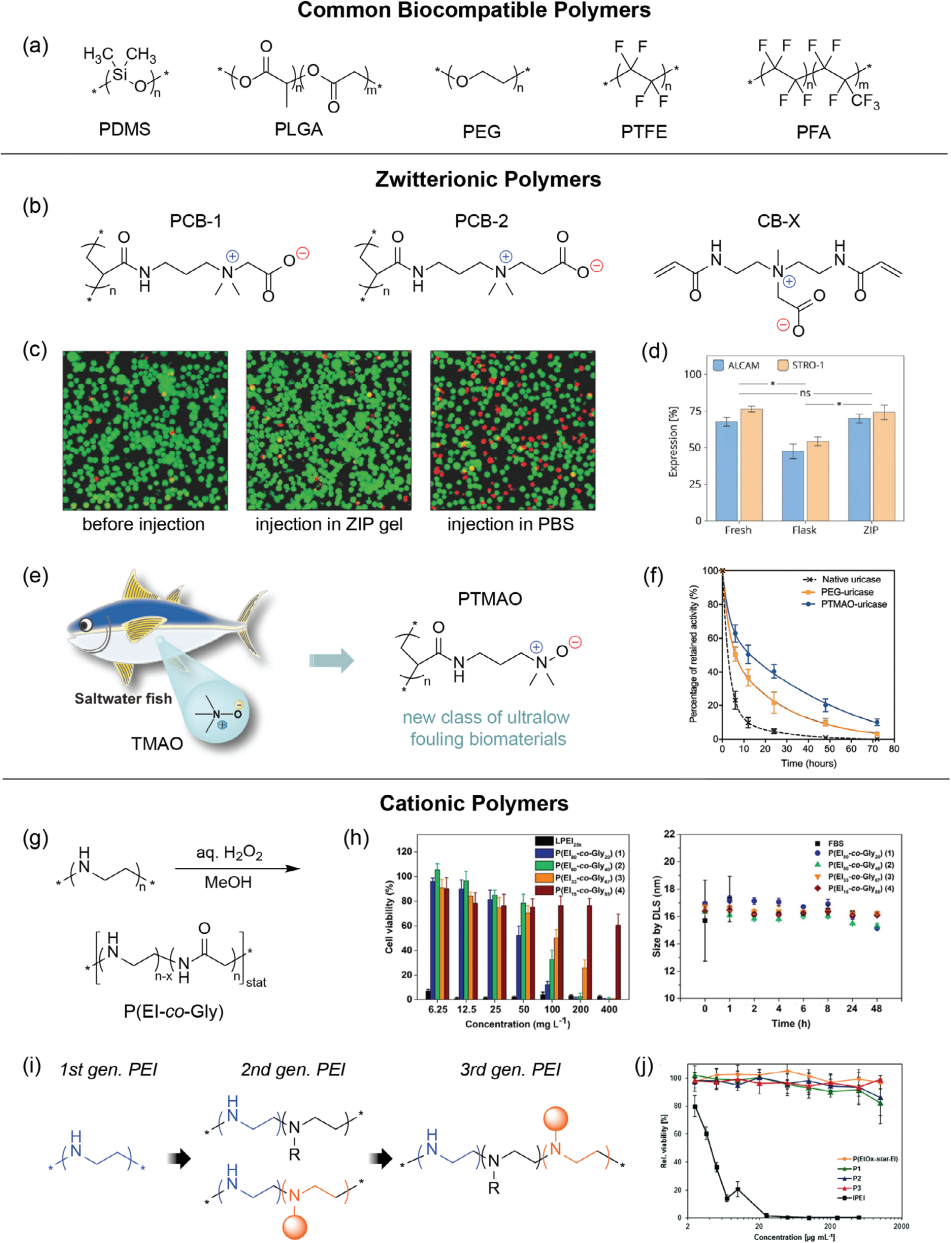
Molecular design for common and emerging biocompatible polymers. a) Chemical structures of biocompatible polymers commonly used in medical applications. b) Chemical structures of the injectable zwitterionic hydrogel platform based on carboxybetaine (CB) polymers and crosslinker. c) LIVE/DEAD stained HEK‐293T cells before and after injection in ZIP gel and PBS control as well as d) expression of multipotency biomarkers ALCAM and STRO‐1 after culture in control flasks and ZIP gels demonstrate improved biocompatibility of the hydrogel. Adapted with permission.^[^ ^109^ ^]^ Copyright 2018, Wiley‐VCH. e) The design of PTMAO is derived from TMAO, a zwitterionic osmolyte in saltwater fishes. f) Pharmacokinetics profile of each uricase protein sample after the third intravenous (IV) injection were determined by measuring the retained activity in mice sera. Adapted with permission.^[^ ^52^ ^]^ Copyright 2019, AAAS. g) Scheme of the oxidation of commercial linear PEI to P(EI‐*co*‐Gly) by H_2_O_2_. h) Cell viability and serum stability of linear PEI as well as P(EI‐*co*‐Gly) copolymers. Adapted with permission.^[^ ^53^ ^]^ Copyright 2015, American Chemical Society. i) Different generations of linear PEI. The multifunctional third generation PEI outperforms first (PEI) and second (single PEI modifications) generations in terms of biocompatibility and biodegradability. j) Relative viability of L929 cells after 24 h incubation with PEI and PEI copolymers at different concentrations. Adapted with permission.^[^ ^50^ ^]^ Copyright 2017, Royal Society of Chemistry.

Retrieved from biological systems, natural polymers, including protein‐ and polysaccharide‐originated polymers (e.g., silk fibroin,^[^ ^85^, ^86^ ^]^ collagen,^[^ ^87^ ^]^ gelatin,^[^ ^88^ ^]^ elastin^[^ ^89^ ^]^) are readily available, inexpensive, and typically biocompatible (i.e., non‐toxic and noninflammatory).^[^ ^90^, ^91^, ^92^ ^]^ More detailed descriptions of various traditional biocompatible and bioresorbable natural polymers and their uses can be found in recent reviews.^[^ ^2^, ^10^ ^]^ However, these naturally‐derived polymers can uncontrollably elicit undesired immunogenic response due to batch‐to‐batch variability or inherent bioactivity.^[^ ^8^, ^93^ ^]^ Synthetic polymers designed to be biocompatible have been widely used in drug delivery,^[^ ^94^, ^95^, ^96^ ^]^ tissue engineering,^[^ ^97^, ^98^ ^]^ and gene transfection.^[^ ^50^, ^99^ ^]^ For example, due to their inertness, low density polyethylene (LDPE) and polydimethylsiloxane (PDMS) have been accepted by the US National Heart, Lung, and Blood Institute as discriminatory tools for validation of both in vitro and in vivo tests in the evaluation of biomaterials.^[^ ^7^ ^]^ Parylene, PLA, PLGA, and PEG are examples of polymers frequently used in implantable electronics and have been approved by the US FDA for clinical use.^[^ ^10^ ^]^ Notably, fluoropolymers have among the best biocompatibility of all plastics due to their typical lubricity, ability to be sterilized, broad temperature tolerance, and minimal chemical reactivity in the body.^[^ ^100^ ^]^ Class VI USP approved fluoropolymers include ethylene tetrafluoroethylene (ETFE), fluorinated ethylene propylene (FEP), perfluoroalkoxy alkane (PFA), polytetrafluoroethylene (PTFE), and polyvinylidene fluoride (PVDF).^[^ ^101^ ^]^ The chemical structures of several common biocompatible polymers are shown in **Figure** **6**a. While there are a number of established and commercial biocompatible polymers, new molecular design allows for synthetic tuning of specific functionalities, enabling a broader range of wearable and implantable electronic applications in different locations of the body and bodily fluids.

#### 4.3.2 Molecular Design of Biocompatible Polymers

The following discussion covers select emerging polymer materials that have been rationally designed to exhibit biocompatible behavior. In designing biocompatible electronics, many traditional hydrophilic polymers can reduce nonspecific protein adsorption, which leads to biofouling, the foreign body reaction, and other adverse biological responses. However, these existing surfaces are often not sufficient in preventing undesirable adhesion of biomolecules. While PEG is widely used and strategies of introducing biocompatibility through grafting PEG side chains have been explored,^[^ ^102^, ^103^ ^]^ PEG is still susceptible to oxidation damage and is less effective in biological media.^[^ ^104^, ^105^ ^]^

##### Zwitterionic Polymers

Over the past few decades, Ratner, Jiang and coworkers have pioneered the development of ultralow fouling zwitterionic materials.^[^ ^104^, ^106^ ^]^ Polymers containing superhydrophilic zwitterionic groups, such as phosphorylcholine (PC), carboxybetaine (CB), and sulfobetaine (SB), which are popular blood‐inert biomaterials, show excellent hydration‐induced nonfouling capability.^[^ ^107^ ^]^ Hydration layers that form on hydrophilic polymer surfaces are known to repel the adsorption of biomolecules with high efficacy, and these zwitterionic materials can bind water more strongly via electrostatics compared to the hydrogen bonding of traditional hydrophilic surfaces.^[^ ^108^ ^]^ Jiang and coworkers reported an injectable and malleable polycarboxybetaine (PCB) hydrogel platform, which shows promise as a tissue filler, drug delivery vehicle, and protective stem cell culture scaffold due to their supportive moduli and tunable viscoelasticity.^[^ ^109^ ^]^ Exclusively CB components were used as they were previously shown to evade the foreign body reaction^[^ ^106^ ^]^ and preserve stem cell multipotency.^[^ ^110^ ^]^ All hydrogels were composed of CB acrylamide monomers with either one‐carbon (PCB‐1) or two‐carbon (PCB‐2) spacers between the charged groups and were crosslinked with CB diacrylamide (CB‐X, 0.01–1 mol%) (Figure 6b). The bulk PCB hydrogels were processed by repeated extrusion into malleable zwitterionic injectable pellet (ZIP) microgels, which can be lyophilized into a powder for mixing with cells or therapeutics. LIVE/DEAD stained HEK‐293T cells suspended in ZIP gel displayed no significant change in cell viability after injection, in contrast with a 25–30% decrease in viability when suspended in the control (PBS only) (Figure 6c). In addition, human mesenchymal stem cells (hMSCs) grown in ZIP scaffolds exhibited high expression of multipotency biomarkers ALCAM and STRO‐1 after 28 days, indistinguishable from the fresh seed population, indicative of restraining hMSC differentiation (Figure 6d).

More recently, Jiang and coworkers introduced a new class of ultralow fouling materials based on trimethylamine *N*‐oxide (TMAO), a zwitterionic osmolyte and protein stabilizer found in saltwater fishes.^[^ ^52^ ^]^ Previous studies showed the hydration capacity, and thus nonfouling property, of zwitterionic polymers increases as the intramolecular distance between the charged sites decreases.^[^ ^111^ ^]^ In TMAO‐derived polymers (PTMAO), charge moieties are directly connected (Me_3_N^+^—O^−^) without a carbon spacer, in contrast with PCB, which contains at least one‐carbon separation (Figure 6e). In vitro fouling tests showed PTMAO resisted fibroblast cell adhesion and protein adsorption in both fibrinogen solution and human blood serum. PTMAO exhibited minimal immunogenicity and extended circulation via subcutaneous and intravenous injections in C57BL/6J mice after being conjugated to highly immunogenic proteins (Figure 6f). The discovery of PTMAO, a fourth class of nonfouling zwitterionic polymers, demonstrates the importance of molecular understanding in designing new biomimetic materials. The advancement of implantable electronics, such as blood‐contacting medical devices, that have prolonged or permanent contact with the body necessitates an encapsulant or coating with properties exhibited by these ultralow fouling materials.

Becker and coworkers introduced a post‐polymerization, surface functionalization strategy for designing antifouling polymers by using radically induced thiol‐ene click reactions to surface derivatize TPUs with zwitterionic thiols.^[^ ^112^ ^]^ TPUs have been widely used for biomedical applications due to their softness and high tensile strength; however, their hydrophobicity can induce undesirable protein adsorption. Spin‐coated TPU thin film substrates with allyl‐ether functionality were submerged in an aqueous solution containing the synthesized zwitterionic thiol followed by treatment with UV light. Protein adsorption experiments by quartz crystal microbalance showed reduced fibrinogen attachment for the surface‐functionalized TPU. The Zwitterion‐TPU also showed a log scale reduction in bacterial adherence. For different bacteria, the polymer resulted in a ≈40 and 50% lower bacterial biomass accumulation compared to its nonfunctionalized controls. Post‐polymerization techniques can be advantageous over copolymerization or blending strategies as it does not alter the bulk property of the material. This reproducible and scalable method for modifying surfaces containing alkene functionalities via the thiol‐ene reaction proved to be an efficient strategy to prepare antifouling surfaces.

##### Cationic Polymers

Cationic polymers, in particular poly(ethylene imine) (PEI), play a crucial role within the field of gene delivery. The cationic ethylene amine can electrostatically interact with the negatively‐charged phosphate groups of DNA and RNA to form polyplexes, which prevent enzymatic degradation and enable DNA/RNA cargo release. The controlled oxidation of linear PEI into poly(ethylene imine‐*co*‐glycine) (P(EI‐*co*‐Gly)) copolymers offers an opportunity for biocompatibility and degradability of an otherwise cytotoxic polymer. Schubert, Yang, Hedrick, and coworkers investigated varying degrees of oxidation of PEI to determine an optimal composition of copolymers for high transfection efficiencies (EI content) along with good cytocompatibility (Gly content) (Figure 6g).^[^ ^53^ ^]^ Additionally, the formed amide groups rendered the copolymers biodegradable by aqueous hydrogen chloride and trypsin. While unmodified linear PEI induces a toxic effect at very low concentrations (<6.25 mg L^−1^), P(EI‐*co*‐Gly_85%_) exhibited ≈80% cell viability at relatively high concentrations (200 mg L^−1^) as well as polymer stability (i.e., no aggregation) after treatment with fetal bovine serum by DLS measurements (Figure 6h).

Further advancing PEIs for non‐viral gene delivery, Traeger, Schubert, and coworkers synthesized a new generation of PEI by post‐polymerization functionalization of partially hydrolyzed poly(2‐ethyl‐2‐oxazoline)s (PEtOx).^[^ ^50^ ^]^ Compared to first (PEI) and second (single PEI modifications) generation PEIs, the new third generation contained varying primary and secondary amines that allowed for small interfering RNA (siRNA) delivery and high transfection efficiencies using plasmid DNA (pDNA) (Figure 6i). Biocompatibility of the copolymer poly(2‐ethyl‐2‐oxazoline‐stat‐ethylene imine) (P(EtOx‐stat‐EI)) and copolymers functionalized with primary amines (P1, P2, P3) was shown by the relative viability of L929 cells after incubation for 24 h at different concentrations according to ISO10993‐5 (Figure 6j). The high cationic charge density of PEI enables a wide variety of applications through structural modification, including human breast cancer cell targeting^[^ ^113^ ^]^ and crossing of the blood‐brain barrier.^[^ ^114^ ^]^ The described modifications to PEI convert the cytotoxic polymer into one that is biocompatible, enabling their potential use in fully biocompatible devices.

Although such cationic polymers have less potential to be used as substrates and encapsulants, fully biocompatible devices that are also biodegradable are important for furthering the development of implantable transient electronics. These recent examples show the importance of considering charge density when designing and modifying the molecular structure of polymers, as post‐polymerization reactions or complexation with non‐toxic moieties can either prevent adverse immune response or enable desired applications in living species. Most polymer‐based electronics currently do not employ charged polymers, leaving a lot of potential for these emerging polymers to be used in implantable electronics. While the typical attractive applications of PEIs in biomedicine cannot be transferred to electronics, PEI has been shown to be an effective dopant in suppressing hole transport and promoting electron transport.^[^ ^115^ ^]^ A small amount of PEI converted ambipolar and p‐type polymer semiconductors into unipolar n‐type semiconductors with improved electron mobility. PEI also serves as a nucleation inducer, shifting the work function of the metal electrodes and enhancing the optoelectronic performance of various device architectures.^[^ ^116^, ^117^ ^]^

## 5 Incorporating Recyclable, Biodegradable, or Biocompatible Polymers into Electronics

In this section, we highlight several examples of polymer‐based electronics that are recyclable, biodegradable, or biocompatible. These electronics range from electrodes to organic field‐effect transistors (OFETs) to electrochemical devices. While these devices consist of various architectures, we will cover polymers by their use as electronic components for encapsulants, substrates, dielectrics, conductors, and semiconductors. Encapsulants, substrates, and dielectrics are typically composed of insulating polymers. There are some considerations to take into account when using polymers that are recyclable, biodegradable, or biocompatible for these components. For example, since encapsulants (if employed) and substrates make up the majority of the device by mass, the recyclability and biodegradation timescales depend heavily on these components. Additionally, human‐friendly implantable devices may only need the encapsulant to be biocompatible, while wearables may only need the substrate to be biocompatible if only one side is interfaced with skin or living tissue. On the other hand, conductors and semiconductors are typically composed of conjugated or doped, conjugated polymers. These electronically‐active polymers have been less studied for their recyclability, biodegradability, and biocompatibility either due to the challenge of incorporating cleavable moieties that maintain conjugation or the numerous amount of existing insulating polymers that are already recyclable, biodegradable, or biocompatible. We will discuss both traditional polymer‐based electronics (i.e., defined either as polymers that are commercial, have been well‐established in literature, or use conventional processes such as secondary/mechanical recycling) as well as emerging polymer‐based electronics. Emerging polymers are defined as polymers that have been discovered and explored in the past decade and, for recycling, as tertiary recyclable polymers that are capable of upcycling. While there has been much advancement in the molecular design of these functional polymers, there are few examples of integration of these new polymer designs into electronics. We will cover recent developments in this area.

### 5.1 Recyclable Electronics

#### 5.1.1 Physical (Secondary) Recycling

**Figure 7:**
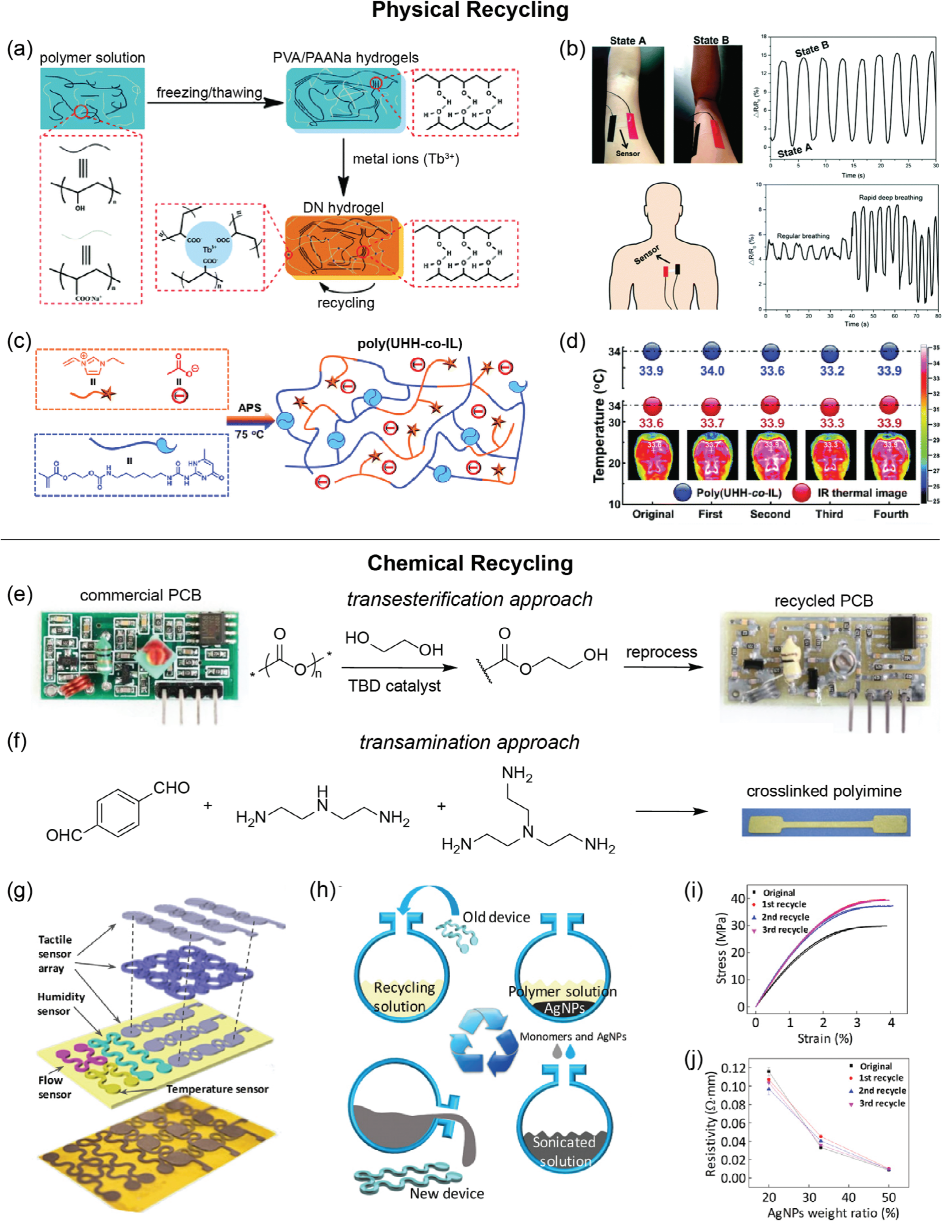
Physically and chemically recyclable polymer‐based electronics. a) Schematic illustration of the preparation and recycling of DN‐hydrogel network structures. The PVA/PAANa hydrogels were formed through three freezing/thawing cycles of polymer solution, and the addition of metal ions effectively enabled crosslinking between Tb^3+^ and its carboxyl groups. b) Photographs and the relative resistance changes of the wearable DN‐hydrogel strain sensor when the forearm was bent and unbent (top) as well as when regular breathing and rapid deep breathing were conducted (bottom). Adapted with permission.^[^ ^24^ ^]^ Copyright 2018, Royal Society of Chemistry. c) Schematic illustration of the recyclable, conductive supramolecular polymer based on an ionic liquid crosslinked by quadruple hydrogen‐bonding interactions. APS: ammonium persulfate d) Forehead temperature measurements via a poly(UHH‐*co*‐IL) sensor recycled four times and IR thermography. Adapted with permission.^[^ ^121^ ^]^ Copyright 2018, Royal Society of Chemistry. e) Small molecule‐assisted dissolution method using ethylene glycol and catalytic triazabicyclodecene (TBD) for the recycling of commercial printed circuit boards. Adapted with permission.^[^ ^123^ ^]^ Copyright 2019, Springer Nature. f) Synthetic scheme for polymerization of the polyimine substrate. g) Schematic illustration (top) and optical image (bottom) of the e‐skin and its multiple sensors made with recyclable polyimine substrate. h) Schematic illustration of an old device soaked in recycling solution and decomposed into oligomers/monomers and silver nanoparticles (AgNPs). After recycling, the solution and AgNPs can be mixed to make new devices. i) Stress–strain curves and j) electrical resistivity measurements of the conductive polyimine films before and after recycling, displaying comparable mechanical properties and electrical performance. Adapted with permission.^[^ ^23^ ^]^ Copyright 2018, AAAS.

Electronics are considered physically recyclable if the degradation and regeneration of a certain material does not involve any externally introduced chemicals. Several of these devices are reprocessed primarily by dissolution techniques.^[^ ^118^, ^119^ ^]^ For example, Zhou, Jin, Liu, and coworkers fabricated flexible strain sensors using recyclable, stretchable, and conductive double network (DN) hydrogels composed of crosslinked poly(vinyl alcohol) (PVA) and poly(acrylic acid sodium) (PAANa) (**Figure** **7**a).^[^ ^24^ ^]^ PVA is crosslinked by crystalline domains, while PAANa is crosslinked by ionic interactions between Tb^3+^ and its carboxyl groups. Due to the recyclability of the individual polymer networks and the conductivity and photoluminescence of Tb^3+^, the resulting DN‐hydrogels have attractive properties for strain sensors. For recycling, DN‐hydrogels were dried and smashed into small pieces. The pieces were then dissolved in water with heating and treated with three freezing/thawing cycles to obtain the recycled material. As the structure was physically crosslinked, the hydrogels were able to be reprocessed by dissolution. The recycled DN‐hydrogel has comparable tensile strength, strain, conductivity, and dynamic rheological properties. The fabricated wearable strain sensors can monitor both large human motions, such as the bending of joints, as well as subtle physiological activities, including swallowing and breathing (Figure 7b). Similarly, Bao and coworkers took advantage of the reversibility of dynamic hydrogen‐bonding to demonstrate the concept of reconfigurable electronics, in which various sensors can be supported on such substrates and cut and reconnected as determined by users.^[^ ^120^ ^]^ The self‐healing supramolecular elastomers were constructed via PDMS oligomers crosslinked by a mixture of strong 4,4‐methylenebis(phenyl urea) (MPU) and weak isophorone bisurea (IU) hydrogen bonds. These multifunctional, physically crosslinked networks further the development of flexible electronics that can be recycled or self‐healed when fractured.

In addition to demonstrating strain sensors using recyclable networks, other physically recyclable electronic devices such as thermal sensors were also demonstrated. Wang and coworkers developed a recyclable and conductive thermal‐sensing device based on an ionic liquid crosslinked by dynamic quadruple hydrogen bonds.^[^ ^121^ ^]^ Copolymerization of a monomer functionalized with 2‐ureido‐4‐[1H]pyrimidinone (UPy) and a conductive vinyl ionic liquid 1‐vinyl‐3‐ethyl‐imidazolium acetate ([VEIm][Ac]) resulted in supramolecular poly(UHH‐*co*‐IL) (Figure 7c). This supramolecular polymer was easily molded into an electronic thermometer with customizable size and shape, serving as a reliable approach to monitor body temperature. In addition, the fabricated thermal sensor showed good reprocessability. With simple grinding and hot pressing the small, fragmented pieces at 90 °C for 2 h, the device was regenerated four times while retaining the same thermal‐sensing performance. Temperature measurements proved to have excellent accuracy, which was verified by IR thermography, even after four recycling cycles (Figure 7d). After use of the customizable sensor for one individual, the material can thus be recycled and remolded to fit someone else. This polymer system combined supramolecular hydrogen‐bonding interactions with ionic liquids, demonstrating a new strategy for recyclable electronic sensors.

These mechanical recycling processes of dissolution and heating have also been employed in assembling polymeric solar cells,^[^ ^118^ ^]^ supercapacitors,^[^ ^119^ ^]^ and conductive adhesives.^[^ ^122^ ^]^ However, they do not return the polymers to their original monomers and simply allow for reprocessing through the breaking of the polymer crosslinks, limiting the types of polymeric materials that are able to be used in electronics. Although the reprocessability of the discussed devices in water and relatively low temperatures (90 °C) enables the ease of recycling, it also results in the inability of devices to be used in a wide range of environments. The mechanical properties of these materials and integrated devices need to be further improved in terms of strength and resistance to external mechanical forces for more robust but also recyclable electronic devices. While these structures’ physical crosslinks, ionic interactions, and hydrogen‐bonding interactions facilitated their recyclability, other types of chemistries such as dynamic covalent bonds can be explored to produce a broader range of recyclable materials for device applications.

#### 5.1.2 Chemical (Tertiary) Recycling

Contrasted with physical (secondary) recycling, chemical (tertiary) recycling forms a separate category in which the depolymerization and regeneration of a material are initiated by chemical reagents, and in most cases, conducted in recycling solutions. While many different chemical recycling processes (e.g., catalysis, acid, base, etc.) have been explored for bulk polymers, comparatively less work has been done in implementing them into electronics recycling. Printed circuit boards (PCBs) are an integral part of any electronic product, account for a large percentage of electronic waste, and are commonly composed of fiberglass epoxy composites, electronic components, and various additives. The waste PCBs have residual value due to the presence of high‐grade precious metals (Au, Ag, Cu, Pd, Ta, etc.). Therefore, the ability to recycle PCBs are important for both environmental protection and economic efficiency. The key step lies in degrading the thermoset organic composites and separating them from electronic components (i.e., high‐grade metals). While mechanical recycling techniques (e.g., grinding, magnetic separation) are currently widely used, these methods do not efficiently recover the metals due to the loss of metal during separation. In a recent report, Qi, Wang, and coworkers employed a small molecule‐assisted approach to dissolve thermosetting polymers containing ester groups and recycle electronic components from PCBs.^[^ ^123^ ^]^ This strategy effectively can recycle a wide range of commercial PCBs, including those made from epoxy‐anhydride or polyester resin substrates. The recycling solution, which was composed of ethylene glycol and catalytic triazabicyclodecene (TBD) in NMP, facilitated transesterification reactions that dissolved the epoxy polymer in 6 h at 180 °C (Figure 7e). The depolymerization of the substrate bonding layer allowed for easy separation of the electronic components and glass fibers from the thermoset resin, achieving efficient material recovery. Additionally, the chemical recyclability of polymers containing ester groups through recycling solutions has the potential to be expanded to other device architectures.

Besides utilizing transesterification reactions, transamination can also be used to chemically recycle a dynamic covalent network‐based nanocomposite for electronic skin (e‐skin) applications. Zhang, Xiao, and coworkers reported a healable, malleable, and fully chemically recyclable e‐skin that mimics functionalities and mechanical properties of natural skin.^[^ ^23^ ^]^ The thermoset‐based e‐skin, composed of a polyimine substrate, was doped with conductive silver nanoparticles (AgNPs), enabling the realization of tactile, temperature, flow, and humidity sensing capabilities (Figure 7f,g). The e‐skin was fully recycled at room temperature by soaking the device in a recycling solution composed of the same reagents used for polyimine synthesis (i.e., ethanol and triamine) (Figure 7h). By first introducing an excess of free primary amines, transamination reactions led to increased end groups within the matrix, reducing the molecular weight and solubilizing the device. The oligomers/monomers and AgNPs which were then used to fabricate a new, functional device. Compared with the previous example of recycling through transesterification, which required catalysts and high temperatures, this transamination strategy effectively dissolved the polyimine‐based device at room temperature without the need of an additional catalyst. In terms of mechanical properties, after three cycles, the Young's modulus and tensile strengths increased by ≈25%, likely due to greater crosslinking density from using recycled oligomers as the starting materials instead of monomers (Figure 7i). Potentially, the relative ratios of different components in the recycling solution can be tuned to obtain identical mechanical properties. On the other hand, the recycling process did not show noticeable influences on electrical performance (Figure 7j). In addition, the malleability of the e‐skin with moderate heating (60 °C) allowed for conformal wearable devices. With good mechanical biocompatibility and recyclability, this next‐generation technology proved to be both human‐ and eco‐friendly and has potential for applications in robotics, prosthetics, health care, and the human‐computer interface. While these works are a good start in fabricating recyclable electronics, there is a need for the integration of new recyclable chemistries that can be applied to different polymer systems, which span a broad range of mechanical and electrical properties. The discussed approaches used in designing emerging recyclable polymers have great potential to be implemented into future electronic devices for environmental sustainability.

### 5.2 Biodegradable Electronics

#### 5.2.1 Traditional Polymer‐Based Devices

Most of the research on developing biodegradable organic electronics are focused on incorporating biodegradable insulating polymer substrates and dielectrics into electronic devices rather than active materials (i.e., semiconductor, conductor). Naturally occurring polymers including silk fibroin,^[^ ^37^, ^124^, ^125^, ^126^ ^]^ cellulose,^[^ ^127^ ^]^ and chitosan^[^ ^92^, ^128^ ^]^ have been widely used as substrate and dielectric components. As substrates typically constitute the majority of devices by mass, with micrometer‐scale thicknesses compared to other components that are on the nanometer‐scale, substrates predominantly dictate the degradation behavior of most devices. Silk fibroin, which is enzymatically degradable, has been explored as substrates due to its highly tunable degradation rate in water. Wallace and coworkers used a silk fibroin‐polypyrrole (SF‐PPy) film cathode coupled with a bioresorbable Mg alloy anode in PBS electrolyte to demonstrate a partially biodegradable Mg–air bioelectric battery.^[^ ^37^ ^]^ These biodegradable energy sources would be crucial to developing implantable devices that would disappear without surgical removal. PPy was chemically coated onto the silk fibroin substrate, which was water‐vapor annealed to reduce *β*‐sheet content for a rapid biodegradation rate. The film degraded in a buffered protease XIV solution (1.0 mg mL^−1^), with a weight loss of 82% after 15 days. Although the biobattery was only partially biodegradable, the authors note that the residual materials have the potential to be eliminated by renal excretion, phagocytosis, and/or endocytosis. However, in vivo and biocompatibility studies were not conducted for this system.

As previously mentioned, the lack of batch control when using natural materials for electronics can lead to undesired bioactivity. Due to this issue, biodegradable synthetic polymers, such as PVA,^[^ ^34^, ^42^, ^43^, ^129^, ^130^ ^]^ PLA,^[^ ^42^, ^129^, ^131^ ^]^ and PLGA^[^ ^34^, ^43^, ^129^ ^]^ have been extensively used in conjunction as substrate and dielectric materials for transient electronics. In 2010, our group demonstrated one of the first fully bioresorbable organic TFTs, selecting PLGA and PVA to be used as the substrate and gate dielectric, respectively, with a small‐molecule semiconductor and gold source/drain electrodes.^[^ ^34^ ^]^ Up to this point, achieving fully transient devices was largely unexplored and had proved to be challenging due to the lack of studies on biodegradable semiconductors and conductors. Although the biodegradation of the small molecule was not explicitly studied, degradation mechanisms for melanin could potentially be expanded to the semiconductor.^[^ ^132^, ^133^ ^]^ Exposing the device to citrate buffer caused the active layer to delaminate from the dielectric in less than 2 days, leading to irreversible loss of functionality. The PLGA substrate, which composed 99.89% of the total mass of the device, resisted degradation for 30 days, after which rapid significant water uptake and mass loss were observed. Almost a decade later, the same synthetic polymers (e.g., PVA, PLA, PLGA) are still widely used as electronics components. For example, Fan, Wang, Li, and coworkers employed a PVA/PBS hydrogel and PLA substrate and nanopillar arrays along with a zinc oxide nanoporous layer in developing a biodegradable capacitor as an energy storage unit for life‐time implantation.^[^ ^42^ ^]^ Similarly, immersing the capacitor in PBS at 37 °C resulted in bulk degradation after 3 months.

**Figure 8:**
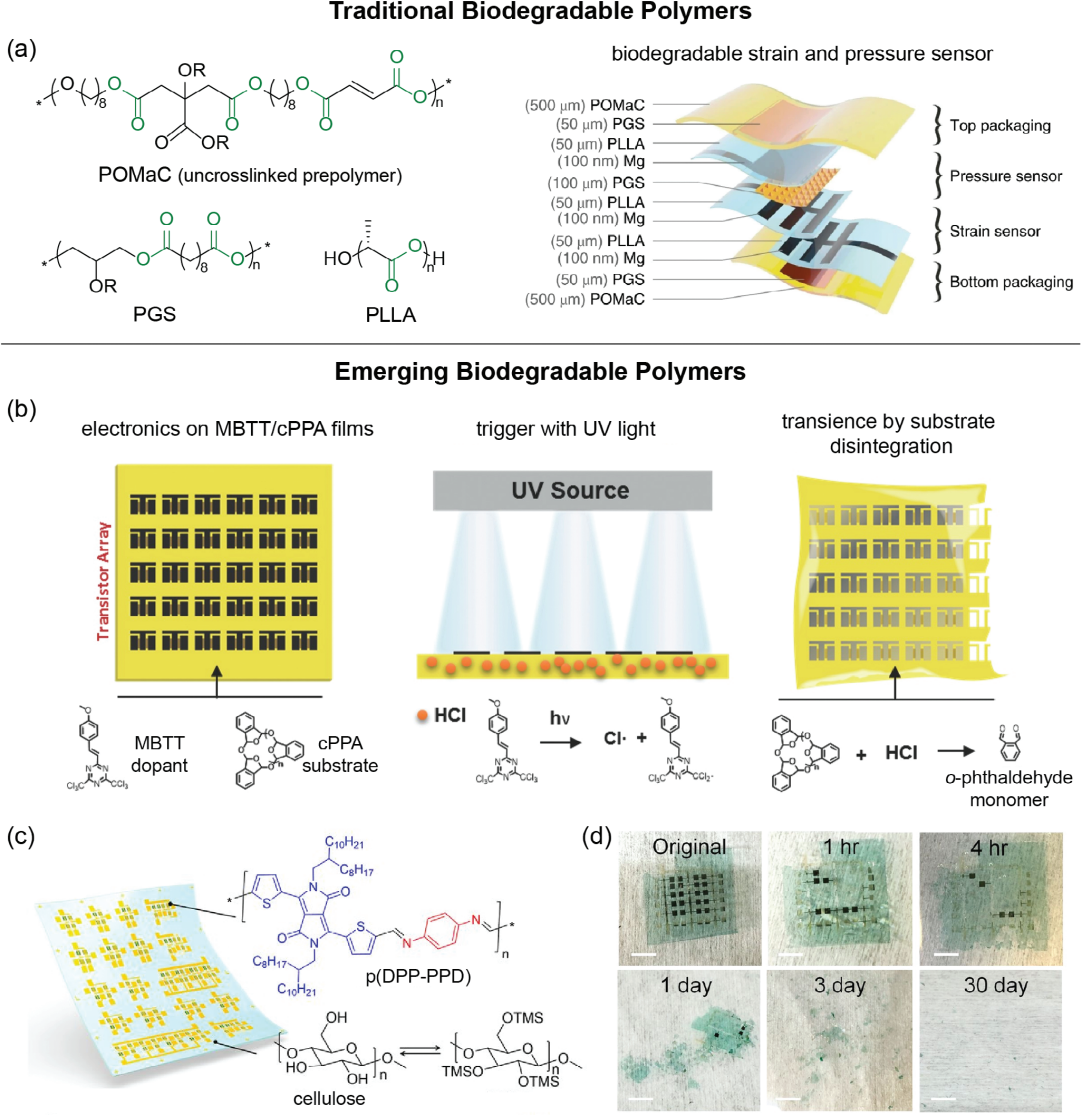
Biodegradable polymer‐based electronics. a) Chemical structures and materials for the assembly of the fully biodegradable strain and pressure sensor. PGS is used as a dielectric layer in the pressure sensor and as a stretchable non‐sticking layer in the strain sensor. POMaC is used for the strain sensor and packaging, while PLLA is the substrate layer for the Mg electrodes. Adapted with permission.^[^ ^134^ ^]^ Copyright 2018, Springer Nature. b) Schematic illustration of the photoinduced transience of electronics on MBTT/cPPA substrates. The generated HCl depolymerized the cPPA substrate and degraded the Mg electrodes. Adapted with permission.^[^ ^40^ ^]^ Copyright 2014, Wiley‐VCH. c) Device using disintegrable polymers p(DPP‐PPD) as the active material and cellulose as the substrate. d) Photographs of the device at various stages of disintegration over 30 days. Adapted with permission.^[^ ^3^ ^]^ Copyright 2017, National Academy of Sciences.

Along with PGS, poly(octamethylene maleate (anhydride) citrate) (POMaC) is another biodegradable elastomer commonly used for tissue engineering and electronic applications. Both PGS and POMaC have been extensively studied for their biocompatibility and biomedical use. Our group has taken advantage of these human‐friendly elastomers by using them alongside biodegradable metal Mg and substrate PLLA for stretchable strain and pressure sensors for orthopedic application^[^ ^134^ ^]^ as well as flexible arterial‐pulse sensors for the wireless monitoring of blood flow.^[^ ^135^ ^]^ The stretchable strain and pressure sensor was fabricated by sandwiching Mg evaporated on top of PLLA with PGS and POMaC dielectric and packaging layers (**Figure** **8**a). A similar design with the addition of polyhydroxybutyrate/polyhydroxyvalerate (PHB/PHV) packaging layers was used for the fabrication of the arterial‐pulse sensor. The stiffer PHB/PHV layer contacted the surrounding muscles, producing a device that was more sensitive to artery expansion than body movement. The backbone ester groups in POMaC permit its hydrolyzability, albeit with slower degradation rates than all other device components (i.e., PGS dielectric, PLLA insulating spacers, PHB/PHV packaging layers). Outside of these conventional synthetic and naturally occurring polymers, there has not been much further development on new types of biodegradable materials for transient electronics. It is crucial to diversify the types of available biodegradable device components to achieve a broad range of degradation rates and conditions for the design of electronics to fit desired applications. Diversification would enable both eco‐friendly and implantable electronics to access degradability in the wide variety of environments in the ecosystem and human body. The described transient devices^[^ ^34^, ^37^, ^42^, ^134^, ^135^ ^]^ are all examples of (type I) partial degradation, in which the electronic components can disintegrate without full chemical breakdown. Although not a large part of the weight the device, undegraded active materials could cause undesired immune response. Recently, demonstrations of electronics that incorporate emerging polymer chemistries and new biodegradable molecular design have been reported,^[^ ^136^, ^137^ ^]^ achieving (type II) complete degradation and expanding the library of biodegradable polymers for device applications.

#### 5.2.2 Emerging Polymer‐Based Devices

While traditional biodegradable polymer‐based devices cover naturally‐derived, commercial, and established polymers in the field, this section will discuss polymer‐based devices that have emerged in the past decade and incorporate new synthetic design. The demonstrated OFETs made from traditional natural polymer dielectrics typically display relatively high threshold voltages that can only operate with gate voltages (*V*_GS_) ≥ 5 V. Majewski and coworkers aimed to fabricate low threshold voltage OFETs that can be operated at *V*_GS_ ≤ 3 V using almond gum naturally derived from almond trees.^[^ ^138^ ^]^ These low voltage OFET devices have potential for use as eco‐friendly, disposable sensors or throwaway, low‐end electronics. Instead of using naturally occurring biopolymers for device components, polymers derived from biorenewable resources may be a more effective way of achieving sustainability while maintaining synthetic control. Most of the natural polymers proposed for dielectric materials have a high hysteresis caused by a large number of polar groups or a low electrical breakdown strength due to their typically not dense structure. Kim and coworkers sought to overcome these problems by using poly‐methacrylated tannic acid (PMTA), which was derived from natural tannic acid extracted from plants, for the realization of naturally degradable crosslinked dielectric materials.^[^ ^139^ ^]^ The densely‐crosslinked structure eliminated hysteresis and advanced electrical breakdown strength, allowing for long‐term stability in ambient environment. At 35 °C, the fabricated TFT was fully decomposed in 8 days in pH 7.4 PBS buffer and in 19 days in a 3.5 wt% sodium chloride solution, mimicking seawater. The PMTA dielectric, synthesized in one step from a bioderived resource, opens opportunity for the reduction of environmental pollution caused by e‐waste through controlled biodegradation in natural environmental conditions.

Previous efforts in transient electronics focused on devices submerged in biofluid or aqueous solution, resulting in a large dependence on the dissolution rate of the materials for degradation. Solution‐based degradation also largely limits transience to biological applications. As substrates generally determine the overall degradation behavior of devices, the use of triggerable or stimuli‐responsive substrates unlocks a new avenue for precise control over the lifetime of the device and expands the current field to include more environmental biodegradation methods. Metastable or self‐immolative polymers, which can be rapidly depolymerized by external stimuli (e.g., humidity, heat, light), are promising candidates to expand the applications of transient devices. Among the metastable polymers, cPPA is ideal due to its low ceiling temperature, which was demonstrated by Moore and coworkers to achieve thermally recyclable materials.^[^ ^22^ ^]^ Thus, Moore, Rogers, White, and coworkers employed cPPA as a substrate and encapsulant with a photo‐acid generator additive to fabricate FETs, diodes, and resistors.^[^ ^40^ ^]^ The phototriggerable degradation of cPPA was demonstrated through use of 2‐(4‐methoxystyryl)‐4,6‐bis(trichloromethyl)‐1,3,5‐triazine (MBTT), which generated hydrochloric acid to react with the acetal backbone of cPPA upon exposure to UV light (Figure 8b). The degradation rate was modified by altering the amount of MBTT and UV exposure. The use of light as a biodegradation method allows for greater control of the degradation of implantable electronics, unlike dissolution and hydrolysis processes which many biological environments allow for. On‐demand photodegradation offers new avenues for use as biomedical diagnostics and remote environmental sensors.

To attain type II materials for organic electronics, the active component must also be biodegradable. Conjugated polymers have shown much promise to be used as biodegradable semiconductors in TFTs; however, until recently, there were not any reports of totally degradable conjugated polymer‐based electronics. Lipomi and coworkers designed stretchable and biodegradable semiconducting block copolymers based on semiconducting DPP and insulating PCL blocks for OFETs.^[^ ^140^ ^]^ Copolymers containing only 10 wt% of DPP had the same field‐effect mobility as neat DPP. The PCL segments in a 50 wt% DPP copolymer were completely degraded in 0.5 m NaOH in 3 days; however, they only degraded to ≈50% of their original content in PBS at physiological temperature after 12 weeks. Although the insulating component decomposed, the semiconductor component did not degrade in PBS after 12 weeks other than an observed 6% reduction in absorbance peak ratio by UV–vis spectroscopy. Our group demonstrated, for the first time, completely degradable type II semiconducting polymers based on reversible imine bonds, which maintain conjugation along the polymer backbone but are concurrently acid‐labile. After immersing the semiconductor in 1% acetic acid in water for 10 days, the absorption spectrum and solution color were both similar to that of the pure monomer. Fully disintegrable and biocompatible TFTs were fabricated from a natural cellulose substrate, biodegradable iron electrodes, and imine‐based p(DPP‐PPD) semiconductors (Figure 8c).^[^ ^3^ ^]^ The prepared devices were completely degradable in a pH 4.6 buffer solution (containing 1 mg mL^−1^ cellulase) within 30 days (Figure 8d). These conditions are milder than household vinegar and gastric acid in the human stomach (pH < 3.5). Further, stretchability was imparted on these fully degradable semiconductors through the blending of p(DPP‐PPD) with a fully degradable elastomer based on PCL (e‐PCL) to result in phase‐segregated semiconductor nanofibers.^[^ ^4^ ^]^ In addition to biodegradability and stretchability, other functionalities such as self‐healing and stimuli‐responsiveness can also be incorporated into these synthetic polymer‐based electronic devices through rational molecular design.^[^ ^141^ ^]^ This property tunability makes polymer‐based systems a promising platform for unrealized skin‐inspired functionalities to meet unmet challenges in the environment and human health as well as have opportunity for use as untraceable electronics in security and defense applications.

### 5.3 Biocompatible Electronics

#### 5.3.1 Traditional Polymer‐Based Devices

**Figure 9:**
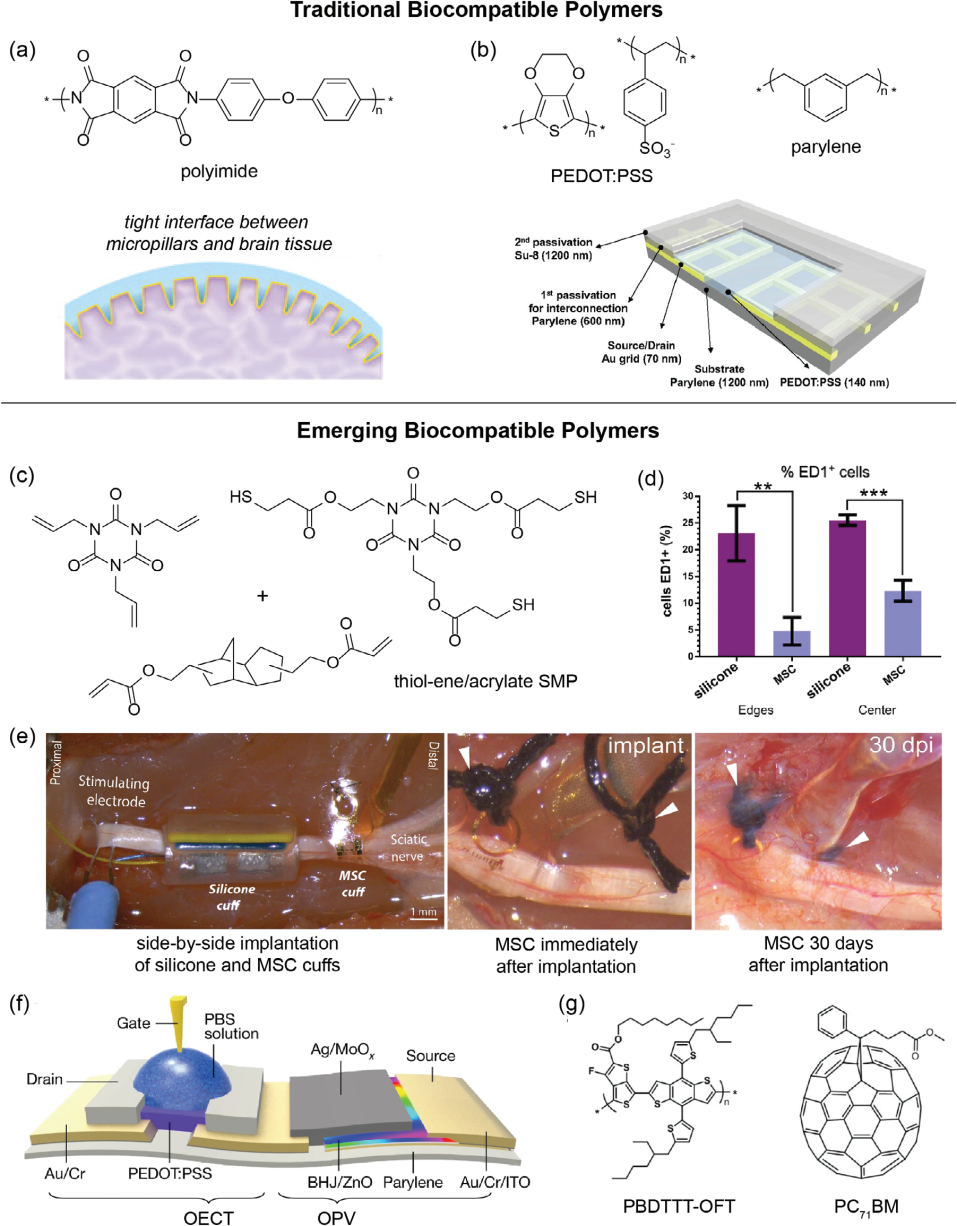
Biocompatible polymer‐based electronics. a) Chemical structure and schematic illustration of the polyimide micropillars tightly interfacing brain tissue. Adapted with permission.^[^ ^148^ ^]^ Copyright 2019, John Wiley and Sons. b) Chemical structures of PEDOT:PSS and parylene, which were used as the active material and substrate, respectively, of the OECT. Adapted with permission.^[^ ^152^ ^]^ Copyright 2017, National Academy of Sciences. c) Chemical structures for the polymerization of thiol‐ene/acrylate SMPs. d) The number of activated macrophages was significantly reduced in the sciatic nerve with MSC electrode. e) Photographs of the in vivo setup showing side‐by‐side implantation of the silicone and MSC electrodes, MSC device immediately after placing, and MSC device 30 days after implantation. The electrode and sutures (arrowheads) are visible through the fibrotic scar. Adapted with permission.^[^ ^49^ ^]^ Copyright 2018, Springer Nature. f) Schematic illustration of a self‐powered device by integration of an OPV with an OECT. g) Chemical structures of PBDTTT‐OFT and PC_71_BM, which were used as the photoactive layer in the OPV. Adapted with permission.^[^ ^155^ ^]^ Copyright 2018, Springer Nature.

Many existing biocompatible electronics are based on easily accessible, widely used, and established polymers, such as PDMS, poly(3,4‐ethylenedioxythiophene) (PEDOT), polypyrrole (Ppy), and poly(3‐hexylthiophene) (P3HT), for use in electrodes, organic field‐effect transistors (OFETs), and electrochemical devices.^[^ ^142^, ^143^, ^144^, ^145^ ^]^ Single electrodes and microelectrode arrays can be implanted in the brain to record and stimulate electrical signals, displaying promise in diagnosing and treating those suffering from epilepsy, Parkinson's disease, and other neurological disorders. Flexible electrodes based on polymer substrates are advantageous over commonly used silicon electrodes, which are much stiffer than brain tissue and eventually lead to chronic inflammation and scarring. As a transparent elastomer, PDMS is often employed as encapsulants and scaffolds for electrodes, imparting both flexibility and biocompatibility.^[^ ^48^, ^146^, ^147^ ^]^ However, the modulus of PDMS is in the MPa range, which is still orders of magnitude higher than human organs such as the brain. This mechanical incompatibility may cause shear force at the electrode‐tissue interface, limiting the stability of signal recording. Li, Fang, and coworkers fabricated a flexible micropillar electrode array (μPEA) based on biocompatible polyimide that readily integrates with the surface of a rat cerebral cortex for in vivo neural activity recordings with a high signal‐to‐noise ratio.^[^ ^148^ ^]^ By using PDMS as a template, micropillars were patterned onto a polyimide substrate, allowing for a tight electrode‐neural interface through engulfment of the micropillars by neural tissues (**Figure** **9**a). Although these polyimide‐based micropillars ensure tight interfacing, the actual modulus of the new surface structure was not measured, and in vivo recordings were only conducted over four weeks.

PEDOT is a well‐known conjugated polymer in the field of bioelectronics for its mechanical flexibility, stability, and high conductivity. The ionomer mixture poly(3,4‐ethylenedioxythiophene) polystyrene sulfonate (PEDOT:PSS) is widely used in wearable bioelectronic devices, electrophysiology sensors, and implantable electronics.^[^ ^149^, ^150^, ^151^ ^]^ Someya and coworkers developed a transparent, ultra‐flexible, and active multielectrode array (MEA), in which the active layer of the organic electrochemical transistor (OECT) is composed of PEDOT:PSS, while parylene makes up the substrate and encapsulant (Figure 9b).^[^ ^152^ ^]^ The MEA enables spatial mapping of electrocorticogram electrical signals for an optogenetic rat, allowing for a neural network system with direct light stimulation. Such established polymers are used commonly as substrates and encapsulants and less commonly as active components in devices. To expand the spectrum of accessible fully biocompatible devices, new polymers with additional functionalities have been explored in recent years and select emerging devices will be discussed in the next section.

#### 5.3.2 Emerging Polymer‐Based Devices

In fabricating the next generation of biocompatible devices, new polymers need to be designed with emerging, unrealized functionalities (e.g., conformal, self‐powered, electronically‐active) to allow for a wider range of biomedical electronic applications that interface with the human body. Peripheral nerve interfaces (PNIs) connect the human peripheral nervous system with electronic devices for electrical stimulation. Silicone, or polysiloxane, nerve cuff electrodes are often implanted on large somatic nerves as PNIs, but their self‐closing mechanism requires thick walls (200–600 µm), resulting in fibrotic tissue growth around and inside the device. Romero‐Ortega and coworkers reported thiol‐ene/acrylate shape memory polymers (SMP) for the fabrication of thin film multi‐electrode softening cuffs (MSC) for the signal recording and stimulation of rat sciatic and pelvic nerves (Figure 9c).^[^ ^49^ ^]^ Thiol‐ene/acrylate SMPs have previously been shown to soften from ≈1800 MPa at room temperature to 41 MPa at 37 °C, which is ideal for use as softening substrates by being stiff during insertion yet soft in vivo.^[^ ^153^, ^154^ ^]^ While the SMP is less soft than traditional silicone (1–50 MPa), the flexural forces are much lower due to its 30 µm thickness. When implanted side‐by‐side and compared after 30 days, the MSC devices displayed significantly less inflammation, indicated by a 70–80% reduction in ED1 positive macrophages and 54–56% less fibrotic vimentin immunoreactivity (Figure 9d). It is important to note that although the MSC device evoked reduced fibrosis compared to their silicone counterpart, these materials still showed a visible fibrotic scar after 30 days and only last for a certain amount of time before suffering from adverse effects of long implantation (Figure 9e). Evaluation of this MSC in chronic studies are needed in order to confirm its functionality over long periods of time, conditions that would more closely resemble potential clinical applications.

Further advancing biocompatible electronics, devices are desired to be self‐powered for the detection of physiological signals without the need for an external power supply or bulky connecting wires. Fukuda, Tajima, Someya, and coworkers realized self‐powered ultra‐flexible devices that can measure biometric signals by integrating OECTs used as sensors with organic photovoltaic (OPV) power sources (Figure 9f).^[^ ^155^ ^]^ Such biocompatible OECTs have received much attention in the field of bioelectronics for their applications in ion sensing, enzymatic sensing, and electrophysiology due to their intimate interfacing with biological components.^[^ ^156^ ^]^ For the OPV, a bulk heterojunction composed of poly[4,8‐bis(5‐(2‐ethylhexyl)thiophen‐2‐yl)benzo[1,2‐b;4,5‐b′]dithiophene‐2,6‐diyl‐alt‐(4‐octyl‐3‐fluorothieno[3,4‐b] thiophene)‐2‐carboxylate‐2‐6‐diyl] (PBDTTT‐OFT) and [6,6]‐phenyl‐C71‐butyric acid methyl ester (PC_71_BM) was used as the photoactive layer (Figure 9g). Their demonstrated cardiac sensor was attached to the exposed surface of a rat heart and electrocardiographic (ECG) signals were measured with high signal‐to‐noise under an LED light. This work integrated ultra‐flexible organic power sources with functional electronic devices for precise and continuous data acquisition for the first time to produce conformal sensors for ECG applications. Additionally, LED light therapy, which can penetrate the skin at varying depths and does not contain harmful ultraviolet rays, has been popularized as a noninvasive treatment and could potentially be expanded to serve as an energy source for biocompatible OPVs in the body.

Many other non‐traditional polymers have been used in biocompatible polymer‐based electronics for a wide variety of applications in recent years. Lanzani, Caironi, and coworkers used semiconductors P3HT, poly[2,5‐bis(2‐decylnonadecyl) pyrrolo[3,4‐c]pyrrole‐1,4‐(2H,5H)‐dione‐(E)‐1,2‐di(2,2′‐bithiophen‐5yl)ethene] (29‐DPP‐TVT), and poly{[*N*,*N*′‐bis(2‐octyldodecyl)‐naphthalene‐1,4,5,8‐bis(dicarboximide)‐2,6‐diyl]‐alt‐5,5′‐(2,2′‐bithiophene)} (P(NDI2OD‐T2)) in OFETs on tattoo‐paper for organic edible electronics.^[^ ^157^ ^]^ Ingestible electronics have potential as therapeutic and diagnostic tools as well as food‐compatible electronic tags that can “smart” track goods along the distribution chain. Ingestible electronics serve as a unique area of biocompatible devices, in which the materials only stay in the human body for the duration of digestion or phagocytosis and thus, only need to be biocompatible for a definite amount of time.^[^ ^158^ ^]^ Another attractive application is for controllable drug release using biocompatible organic electronic ion pumps. Chen, Yan, and coworkers demonstrated P3HT has a switchable permeability in aqueous solutions under a low bias voltage which can be used in organic devices for the controlled release of molecules.^[^ ^159^ ^]^ Lastly, as a valid alternative to these conjugated polymers, organic blends of conjugated small molecules and insulating polymers can serve as bioelectronics transducers. Kyndiah and coworkers blended small‐molecule semiconductor 2,8‐difluoro‐5,11‐bis(triethylsilylethynyl)anthradithiophene (diF‐TES‐ADT) with an insulating polymer to realize electrolyte gated OFETs which can stably record the extracellular potential of human pluripotent stem cell derived cardiomyocyte cells (hPSCs‐CMs).^[^ ^160^ ^]^ Although commercial biocompatible polymers have been widely used in biocompatible electronics, they are typically insulating, only allowing for use as encapsulants and substrates. Besides PEDOT:PSS, biocompatible conjugated polymers have been less explored in electronics until the past few decades. These emerging electronically‐active polymers allow for higher performance when used as active electronic components in OFETs, OECTs, and OPVs as well as additional functionalities such as being conformal and self‐powered.

## 6 Conclusions and Outlook

This review provides an overview on the field of electronics that are recyclable, biodegradable, and biocompatible. In attempts to organize reported literature, we clarified the respective definitions of each term in the context of industrial standards. Since these materials will be interacting with the human body and environment, it is important to understand the classification for real‐life applications. Moreover, this review provides examples of analytical techniques used in reported literature to characterize chemical and mechanical properties for materials which undergo recycling, biodegrade, or interact with biological systems. Standard metrics for characterization currently do not exist, partly because the transformation modes are strongly dependent on the application and intended time of use. The analytical techniques probe at the molecular level (e.g., NMR) to the microscopic level (e.g., UV–vis) to the macroscopic level (e.g., stress–strain curves for mechanical resilience). Next, we provide a perspective on the molecular design rules that are commonly employed for materials that are recyclable, biodegradable, and biocompatible. In addition to established chemistries and materials, we highlighted advances in polymer chemistry which have not be used in electronics. Lastly, our review showcases electronic devices that exhibit an element of recyclability, biodegradability, or biocompatibility.

By adopting the emerging chemistries developed by the polymer community in the past decade, there is an untapped potential to advance electronics to become recyclable, biodegradable, and biocompatible. The limited examples of such recently reported electronics exemplify the unexplored applications accessible by recyclable, biodegradable, or biocompatible materials. While these electronics so far have used well‐known materials, such as PDMS or PGS, we anticipate that the use of new polymer chemistries will enable unrealized functionalities and fine‐tune mechanical control while achieving eco‐ and human‐friendly integration into society. As the components (e.g., encapsulants, substrates, dielectrics, semiconductors, conductors) in electronic devices require the use of polymeric materials with differing chemical and mechanical properties, it is important to consider how emerging chemistries can be utilized for each component to achieve a fully functional, sustainable device. For example, chemically recyclable linear polymers reported by Hillmyer and Hoye enable the regeneration of monomers, and thus, upcycling of polymeric components used in electronics.^[^ ^63^, ^64^ ^]^ These insulating thermoplastics enable the use of substrates or dielectrics that can be recycled from processes (e.g., catalysis, acid, base) other than standard secondary (mechanical) recycling methods. A fully recyclable device, where each component is recycled under different conditions, can be envisioned to allow for the separation of different device components as well as selective recycling or degradation of a target component. Additionally, emerging dynamic covalent chemistries used in recyclable thermosetting materials allow for a wider range of crosslinked polymeric materials with more robust or stable mechanical properties to be used for recyclable electronics.^[^ ^21^, ^26^, ^72^ ^]^ García's strategy of blending conductive nanofillers into recyclable networks further enables these thermosetting materials to be used as electronically‐active components (i.e., semiconductors, conductors).^[^ ^27^ ^]^

Recent examples of biodegradable and biocompatible polymeric materials can also be integrated to advance wearable or implantable device components with new rationally designed functionalities or desired mechanical properties. For example, the living polymerizations of degradable acetal polymers demonstrated by Gutekunst allow for narrow polydispersities and block copolymers, which enable greater mechanical control and synthetic design of substrates or dielectrics.^[^ ^35^ ^]^ These modular enyne monomers can be further tuned to be more hydrophilic to facilitate implantable electronic applications in the body. Moreover, the biodegradable polyester elastomers reported by Sander, Coates, and Hillmyer can serve as alternative stretchable substrates to the commonly used PGS, enabling more robust degradation at physiologically and environmentally relevant temperatures via enzymes.^[^ ^39^ ^]^ Interesting and unexpected labile linkages that maintain backbone conjugation, such as vinylene bonds that undergo enzymatic cleavage, can also be envisioned to be implemented as polymer semiconductors.^[^ ^20^, ^141^ ^]^ In terms of biocompatibility, the non‐fouling zwitterionic materials reported by Jiang and Becker are ideally suited as encapsulants for implantable devices to mitigate undesirable adhesion of biomolecules and adverse immune response.^[^ ^52^, ^109^ ^]^ While the mechanical properties of these non‐fouling polymers have been less studied, they can be further synthetically designed to impart stretchability as the subjection of implantable electronics to repetitive motions demands certain elasticity (>30% strain, as compared to the stretchability of human skin) and fatigue resistance. Additionally, as the emerging biocompatible polymers discussed demonstrate the ability of charged or polar moieties in the polymer structure to enable biocompatibility, the grafting of charged or zwitterionic motifs off semiconducting polymer chains would potentially enable their biocompatibilities.

The expansion of polymer chemistry and synthetic design in recent years provides huge potential for integration into multifunctional electronics. As we continue to advance recyclable, biodegradable, biocompatible polymers and polymer‐based electronics, it is also important to consider potential emerging issues. With the increase of widespread use of biodegradable polymers in society, they can easily become contaminants in a recycle stream if they degrade during reprocessing. Additionally, many polymers and electronics that interface with biological systems only show biocompatibility for a certain amount of time that may not be sufficient for their intended use in real‐life applications. While the examples highlighted enable the integration of emerging recyclable, biodegradable, and biocompatible chemistries into electronics, an even wider range of sustainability can be applied to the next‐generation of electronics by use of biobased or renewable materials and green syntheses, some of which have been demonstrated in this review. Combinatorial libraries of recyclable, biodegradable, and biocompatible polymers could also be designed as tools for future development of the most effective eco‐ and human‐friendly devices.

## Conflict of Interest

The authors declare no conflict of interest.

## References

[1] A. Rahimi , J. M. García , Nat. Rev. Chem. 2017, 1, 0046.

[2] Y. Cao , K. E. Uhrich , J. Bioact. Compat. Polym. 2019, 34, 3.

[3] T. Lei , M. Guan , J. Liu , H.‐C. Lin , R. Pfattner , L. Shaw , A. F. McGuire , T.‐C. Huang , L. Shao , K.‐T. Cheng , J. B.‐H. Tok , Z. Bao , Proc. Natl. Acad. Sci. U. S. A. 2017, 114, 5107. 28461459 10.1073/pnas.1701478114 PMC5441761

[4] H. Tran , V. R. Feig , K. Liu , H.‐C. Wu , R. Chen , J. Xu , K. Deisseroth , Z. Bao , ACS Cent. Sci. 2019, 5, 1884. 31807690 10.1021/acscentsci.9b00850 PMC6891860

[5] I. A. Ignatyev , W. Thielemans , B. Vander Beke , ChemSusChem 2014, 7, 1579. 24811748 10.1002/cssc.201300898

[6] D. K. Schneiderman , M. A. Hillmyer , Macromolecules 2017, 50, 3733.

[7] M. Irimia‐Vladu , Chem. Soc. Rev. 2014, 43, 588. 24121237 10.1039/c3cs60235d

[8] V. R. Feig , H. Tran , Z. Bao , ACS Cent. Sci. 2018, 4, 337. 29632879 10.1021/acscentsci.7b00595 PMC5879474

[9] H. Liu , R. Jian , H. Chen , X. Tian , C. Sun , J. Zhu , Z. Yang , J. Sun , C. Wang , Nanomaterials 2019, 9, 950. 10.3390/nano9070950 PMC6669760 31261962

[10] X. Huang , Bioresorbable Materials and Their Application in Electronics, Cambridge University Press, Cambridge 2017.

[11] A. A. L. Mattina , S. Mariani , G. Barillaro , Adv. Sci. 2020, 7, 1902872. 10.1002/advs.201902872 PMC7029671 32099766

[12] K. Liu , H. Tran , V. R. Feig , Z. Bao , MRS Bull. 2020, 45, 96.

[13] S. M. Al‐Salem , P. Lettieri , J. Baeyens , Prog. Energy Combust. Sci. 2010, 36, 103.

[14] M. Vert , Y. Doi , K.‐H. Hellwich , M. Hess , P. Hodge , P. Kubisa , M. Rinaudo , F. Schué , Pure Appl. Chem. 2012, 84, 377.

[15] J. M. Garcia , M. L. Robertson , Science 2017, 358, 870. 29146798 10.1126/science.aaq0324

[16] A. Brems , J. Baeyens , R. Dewil , Therm. Sci. 2012, 16, 669.

[17] Biocompatibility of Plastics | Zeus, https://www.zeusinc.com/biocompatibility‐of‐plastics/ (accessed: August 2020).

[18] I. S. O. 15270:2008(en) , Plastics — Guidelines for the recovery and recycling of plastics waste , https://www.iso.org/obp/ui/#iso:std:iso:15270:ed‐2:v1:en (accessed: August 2020).

[19] A. P. R. D.® G. Home, https://plasticsrecycling.org/apr‐design‐guide/apr‐design‐guide‐home (accessed: August 2020).

[20] Q. Miao , C. Xie , X. Zhen , Y. Lyu , H. Duan , X. Liu , J. V. Jokerst , K. Pu , Nat. Biotechnol. 2017, 35, 1102. 29035373 10.1038/nbt.3987

[21] L. M. Polgar , M. van Duin , A. A. Broekhuis , F. Picchioni , Macromolecules 2015, 48, 7096.

[22] E. M. Lloyd , H. L. Hernandez , A. M. Feinberg , M. Yourdkhani , E. K. Zen , E. B. Mejia , N. R. Sottos , J. S. Moore , S. R. White , Chem. Mater. 2019, 31, 398.

[23] Z. Zou , C. Zhu , Y. Li , X. Lei , W. Zhang , J. Xiao , Sci. Adv. 2018, 4, eaaq0508. 29487912 10.1126/sciadv.aaq0508 PMC5817920

[24] J. Lai , H. Zhou , M. Wang , Y. Chen , Z. Jin , S. Li , J. Yang , X. Jin , H. Liu , W. Zhao , J. Mater. Chem. C 2018, 6, 13316.

[25] J.‐B. Zhu , E. M. Watson , J. Tang , E. Y.‐X. Chen , Science 2018, 360, 398. 29700260 10.1126/science.aar5498

[26] P. R. Christensen , A. M. Scheuermann , K. E. Loeffler , B. A. Helms , Nat. Chem. 2019, 11, 442. 31011169 10.1038/s41557-019-0249-2

[27] A. Rahimi , A. Herzog‐Arbeitman , J. M. García , Macromol. Mater. Eng. 2019, 304, 1800583.

[28] ASTM International , D5511‐18 Standard Test Method for Determining Anaerobic Biodegradation of Plastic Materials Under High‐Solids Anaerobic‐Digestion Conditions, ASTM International, West Conshohocken, PA 2018,

[29] ASTM International , D5526‐18 Standard Test Method for Determining Anaerobic Biodegradation of Plastic Materials Under Accelerated Landfill Conditions, ASTM International, West Conshohocken, PA 2018,

[30] ASTM International , D6400‐19 Standard Specification for Labeling of Plastics Designed to Be Aerobically Composted in Municipal or Industrial Facilities, ASTM International, West Conshohocken, PA 2019,

[31] ASTM International , D6868‐19 Standard Specification for Labeling of End Items That Incorporate Plastics and Polymers as Coatings or Additives with Paper and Other Substrates Designed to Be Aerobically Composted in Municipal or Industrial Facilities, ASTM International, West Conshohocken, PA 2019,

[32] Packaging waste directive and standards for compostability, https://www.bpf.co.uk/topics/standards_for_compostability.aspx (accessed: September 2020).

[33] 14:00‐17:00, “ISO 14855‐1:2005, https://www.iso.org/cms/render/live/en/sites/isoorg/contents/data/standard/04/21/42155.html (accessed: September 2020).

[34] C. J. Bettinger , Z. Bao , Adv. Mater. 2010, 22, 651. 20217767 10.1002/adma.200902322 PMC2868598

[35] L. Fu , X. Sui , A. E. Crolais , W. R. Gutekunst , Angew. Chem., Int. Ed. 2019, 58, 15726. 10.1002/anie.201909172 PMC7265103 31487416

[36] S. Samanta , D. R. Bogdanowicz , H. H. Lu , J. T. Koberstein , Macromolecules 2016, 49, 1858.

[37] X. Jia , C. Wang , C. Zhao , Y. Ge , G. G. Wallace , Adv. Funct. Mater. 2016, 26, 1454.

[38] H. J. Kim , Y. Reddi , C. J. Cramer , M. A. Hillmyer , C. J. Ellison , ACS Macro Lett. 2020, 9, 96. 10.1021/acsmacrolett.9b00890 35638662

[39] G. X. De Hoe , M. T. Zumstein , B. J. Tiegs , J. P. Brutman , K. McNeill , M. Sander , G. W. Coates , M. A. Hillmyer , J. Am. Chem. Soc. 2018, 140, 963. 29337538 10.1021/jacs.7b10173

[40] H. L. Hernandez , S.‐K. Kang , O. P. Lee , S.‐W. Hwang , J. A. Kaitz , B. Inci , C. W. Park , S. Chung , N. R. Sottos , J. S. Moore , J. A. Rogers , S. R. White , Adv. Mater. 2014, 26, 7637. 25332056 10.1002/adma.201403045

[41] Y. Wang , G. A. Ameer , B. J. Sheppard , R. Langer , Nat. Biotechnol. 2002, 20, 602. 12042865 10.1038/nbt0602-602

[42] H. Li , C. Zhao , X. Wang , J. Meng , Y. Zou , S. Noreen , L. Zhao , Z. Liu , H. Ouyang , P. Tan , M. Yu , Y. Fan , Z. L. Wang , Z. Li , Adv. Sci. 2019, 6, 1801625. 10.1002/advs.201801625 PMC6425441 30937259

[43] X. Peng , K. Dong , C. Ye , Y. Jiang , S. Zhai , R. Cheng , D. Liu , X. Gao , J. Wang , Z. L. Wang , Sci. Adv. 2020, 6, eaba9624. 32637619 10.1126/sciadv.aba9624 PMC7319766

[44] 14:00‐17:00, “ISO 10993‐1:2018, https://www.iso.org/cms/render/live/en/sites/isoorg/contents/data/standard/06/89/68936.html (accessed: August 2020).

[45] General Chapters: <88> BIOLOGICAL REACTIVITY TESTS, IN VIVO, http://www.uspbpep.com/usp29/v29240/usp29nf24s0_c88.html (accessed: August 2020).

[46] Regulation (EU) 2017/745 of the European Parliament and of the Council of 5 April 2017 on Medical Devices, Amending Directive 2001/83/EC, Regulation (EC) No 178/2002 and Regulation (EC) No 1223/2009 and Repealing Council Directives 90/385/EEC and 93/42/EEC (Text with EEA Relevance) 2017.

[47] Regulation (EU) 2017/746 of the European Parliament and of the Council of 5 April 2017 on in Vitro Diagnostic Medical Devices and Repealing Directive 98/79/EC and Commission Decision 2010/227/EU (Text with EEA Relevance) 2017.

[48] S. Beach , S. Grundeen , A. Doyle , L. Theogarajan , Eng. Res. Express 2020, 2, 025025.

[49] M. A. González‐González , A. Kanneganti , A. Joshi‐Imre , A. G. Hernandez‐Reynoso , G. Bendale , R. Modi , M. Ecker , A. Khurram , S. F. Cogan , W. E. Voit , M. I. Romero‐Ortega , Sci. Rep. 2018, 8, 16390. 30401906 10.1038/s41598-018-34566-6 PMC6219541

[50] T. Bus , C. Englert , M. Reifarth , P. Borchers , M. Hartlieb , A. Vollrath , S. Hoeppener , A. Traeger , U. S. Schubert , J. Mater. Chem. B 2017, 5, 1258. 32263594 10.1039/c6tb02592g

[51] X. Jing , H.‐Y. Mi , X.‐F. Peng , L.‐S. Turng , Carbon 2018, 136, 63.

[52] B. Li , P. Jain , J. Ma , J. K. Smith , Z. Yuan , H.‐C. Hung , Y. He , X. Lin , K. Wu , J. Pfaendtner , S. Jiang , Sci. Adv. 2019, 5, eaaw9562. 31214655 10.1126/sciadv.aaw9562 PMC6570511

[53] C. Englert , M. Hartlieb , P. Bellstedt , K. Kempe , C. Yang , S. K. Chu , X. Ke , J. M. García , R. J. Ono , M. Fevre , R. J. Wojtecki , U. S. Schubert , Y. Y. Yang , J. L. Hedrick , Macromolecules 2015, 48, 7420.

[54] X. Jia , C. Qin , T. Friedberger , Z. Guan , Z. Huang , Sci. Adv. 2016, 2, e1501591. 27386559 10.1126/sciadv.1501591 PMC4928905

[55] R. Aguado , M. Olazar , J. San José María , B. Gaisán , J. Bilbao , Energy Fuels 2002, 16, 1429.

[56] G. Celik , R. M. Kennedy , R. A. Hackler , M. Ferrandon , A. Tennakoon , S. Patnaik , A. M. LaPointe , S. C. Ammal , A. Heyden , F. A. Perras , M. Pruski , S. L. Scott , K. R. Poeppelmeier , A. D. Sadow , M. Delferro , ACS Cent. Sci. 2019, 5, 1795. 31807681 10.1021/acscentsci.9b00722 PMC6891864

[57] K. Fukushima , J. M. Lecuyer , D. S. Wei , H. W. Horn , G. O. Jones , H. A. Al‐Megren , A. M. Alabdulrahman , F. D. Alsewailem , M. A. McNeil , J. E. Rice , J. L. Hedrick , Polym. Chem. 2013, 4, 1610.

[58] R. E. Yardley , A. R. Kenaree , E. R. Gillies , Macromolecules 2019, 52, 6342.

[59] A. M. DiLauro , J. S. Robbins , S. T. Phillips , Macromolecules 2013, 46, 2963.

[60] A. M. DiLauro , A. Abbaspourrad , D. A. Weitz , S. T. Phillips , Macromolecules 2013, 46, 3309.

[61] M. Hong , E. Y.‐X. Chen , Nat. Chem. 2016, 8, 42. 26673263 10.1038/nchem.2391

[62] X. Tang , E. Y.‐X. Chen , Chem 2019, 5, 284.

[63] D. K. Schneiderman , M. E. Vanderlaan , A. M. Mannion , T. R. Panthani , D. C. Batiste , J. Z. Wang , F. S. Bates , C. W. Macosko , M. A. Hillmyer , ACS Macro Lett. 2016, 5, 515. 10.1021/acsmacrolett.6b00193 35607243

[64] G. W. Fahnhorst , T. R. Hoye , ACS Macro Lett. 2018, 7, 143. 10.1021/acsmacrolett.7b00889 PMC12277036 35610909

[65] X. Kuang , G. Liu , X. Dong , X. Liu , J. Xu , D. Wang , J. Polym. Sci., Part A: Polym. Chem. 2015, 53, 2094.

[66] X. Chen , M. A. Dam , K. Ono , A. Mal , H. Shen , S. R. Nutt , K. Sheran , F. Wudl , Science 2002, 295, 1698. 11872836 10.1126/science.1065879

[67] D. Montarnal , M. Capelot , F. Tournilhac , L. Leibler , Science 2011, 334, 965. 22096195 10.1126/science.1212648

[68] W. Denissen , G. Rivero , R. Nicolaÿ , L. Leibler , J. M. Winne , F. E. D. Prez , Adv. Funct. Mater. 2015, 25, 2451.

[69] A. Rekondo , R. Martin , A. R. de Luzuriaga , G. Cabañero , H. J. Grande , I. Odriozola , Mater. Horiz. 2014, 1, 237.

[70] P. Zheng , T. J. McCarthy , J. Am. Chem. Soc. 2012, 134, 2024. 22280441 10.1021/ja2113257

[71] M. Röttger , T. Domenech , R. van der Weegen , A. Breuillac , R. Nicolaÿ , L. Leibler , Science 2017, 356, 62. 28386008 10.1126/science.aah5281

[72] P. Shieh , W. Zhang , K. E. L. Husted , S. L. Kristufek , B. Xiong , D. J. Lundberg , J. Lem , D. Veysset , Y. Sun , K. A. Nelson , D. L. Plata , J. A. Johnson , Nature 2020, 583, 542. 32699399 10.1038/s41586-020-2495-2 PMC7384294

[73] J. M. García , G. O. Jones , K. Virwani , B. D. McCloskey , D. J. Boday , G. M. ter Huurne , H. W. Horn , D. J. Coady , A. M. Bintaleb , A. M. S. Alabdulrahman , F. Alsewailem , H. A. A. Almegren , J. L. Hedrick , Science 2014, 344, 732. 24833389 10.1126/science.1251484

[74] M. J. Krause , T. G. Townsend , Environ. Sci. Technol. Lett. 2016, 3, 166.

[75] Y. Wang , Y. M. Kim , R. Langer , J. Biomed. Mater. Res. A 2003, 66, 192. 12833446 10.1002/jbm.a.10534

[76] G. Mabilleau , A. Sabokbar , in Degradation Rate of Bioresorbable Materials, Elsevier, Amsterdam 2008, pp. 145–160.

[77] D. Li , Y. Bu , L. Zhang , X. Wang , Y. Yang , Y. Zhuang , F. Yang , H. Shen , D. Wu , Biomacromolecules 2016, 17, 291. 26682612 10.1021/acs.biomac.5b01394

[78] C.‐H. Whang , K. S. Kim , J. Bae , J. Chen , H.‐W. Jun , S. Jo , Macromol. Rapid Commun. 2017, 38, 1700395. 10.1002/marc.201700395 28833950

[79] R. T. Tran , P. Thevenot , D. Gyawali , J.‐C. Chiao , L. Tang , J. Yang , Soft Matter 2010, 6, 2449. 22162975 10.1039/C001605E PMC3233194

[80] O. Nsengiyumva , S. A. Miller , Green Chem. 2019, 21, 973.

[81] A. M. Jazani , J. K. Oh , Polym. Chem. 2020, 11, 2934.

[82] T. Iwata , Y. Doi , Macromolecules 1998, 31, 2461.

[83] Y. Tokiwa , B. P. Calabia , C. U. Ugwu , S. Aiba , Int. J. Mol. Sci. 2009, 10, 3722. 19865515 10.3390/ijms10093722 PMC2769161

[84] R.‐J. Mueller , Process Biochem. 2006, 41, 2124.

[85] E. Wenk , H. P. Merkle , L. Meinel , J. Controlled Release 2011, 150, 128. 10.1016/j.jconrel.2010.11.007 21059377

[86] J.‐W. Seo , H. Kim , K. Kim , S. Q. Choi , H. J. Lee , Adv. Funct. Mater. 2018, 28, 1800802.

[87] S. A. Sell , M. J. McClure , K. Garg , P. S. Wolfe , G. L. Bowlin , Adv. Drug Delivery Rev. 2009, 61, 1007. 10.1016/j.addr.2009.07.012 19651166

[88] A. Duconseille , T. Astruc , N. Quintana , F. Meersman , V. Sante‐Lhoutellier , Food Hydrocolloids 2015, 43, 360.

[89] C. N. Grover , R. E. Cameron , S. M. Best , J. Mech. Behav. Biomed. Mater. 2012, 10, 62. 22520419 10.1016/j.jmbbm.2012.02.028

[90] W. Xu , M. Xiao , L. Yuan , J. Zhang , Z. Hou , Polymers 2018, 10, 580. 10.3390/polym10060580 PMC6404008 30966614

[91] Y. Chen , L. Yu , B. Zhang , W. Feng , M. Xu , L. Gao , N. Liu , Q. Wang , X. Huang , P. Li , W. Huang , Biomacromolecules 2019, 20, 2230. 31070896 10.1021/acs.biomac.9b00179

[92] L. Wang , Z. Lou , K. Wang , S. Zhao , P. Yu , W. Wei , D. Wang , W. Han , K. Jiang , G. Shen , Research 2020, 2020, 8716847. 32529189 10.34133/2020/8716847 PMC7171591

[93] L. S. Nair , C. T. Laurencin , Prog. Polym. Sci. 2007, 32, 762.

[94] C. Englert , J. C. Brendel , T. C. Majdanski , T. Yildirim , S. Schubert , M. Gottschaldt , N. Windhab , U. S. Schubert , Prog. Polym. Sci. 2018, 87, 107.

[95] W. Chen , S. Zhou , L. Ge , W. Wu , X. Jiang , Biomacromolecules 2018, 19, 1732. 29690764 10.1021/acs.biomac.8b00218

[96] F. Zabihi , P. Graff , F. Schumacher , B. Kleuser , S. Hedtrich , R. Haag , Nanoscale 2018, 10, 16848. 30168550 10.1039/c8nr05536j

[97] A. Dominguez‐Alfaro , N. Alegret , B. Arnaiz , J. M. González‐Domínguez , A. Martin‐Pacheco , U. Cossío , L. Porcarelli , S. Bosi , E. Vázquez , D. Mecerreyes , M. Prato , ACS Biomater. Sci. Eng. 2020, 6, 1269. 33464834 10.1021/acsbiomaterials.9b01316

[98] C. Ning , Z. Zhou , G. Tan , Y. Zhu , C. Mao , Prog. Polym. Sci. 2018, 81, 144. 29983457 10.1016/j.progpolymsci.2018.01.001 PMC6029263

[99] Y. Huang , X. Ding , Y. Qi , B. Yu , F.‐J. Xu , Biomaterials 2016, 106, 134. 27561884 10.1016/j.biomaterials.2016.08.025

[100] H. Teng , Appl. Sci. 2012, 2, 496.

[101] B. J. Henry , J. P. Carlin , J. A. Hammerschmidt , R. C. Buck , L. W. Buxton , H. Fiedler , J. Seed , O. Hernandez , Integr. Environ. Assess. Manage. 2018, 14, 316. 10.1002/ieam.4035 29424474

[102] J.‐F. Lutz , J. Polym. Sci., Part A: Polym. Chem. 2008, 46, 3459.

[103] R. Fukai , P. H. R. Dakwa , W. Chen , J. Polym. Sci., Part A: Polym. Chem. 2004, 42, 5389.

[104] S. Jiang , Z. Cao , Adv. Mater. 2010, 22, 920. 20217815 10.1002/adma.200901407

[105] V. Gaberc‐Porekar , I. Zore , B. Podobnik , V. Menart , Curr. Opin. Drug Discov. Devel. 2008, 11, 242. 18283612

[106] L. Zhang , Z. Cao , T. Bai , L. Carr , J.‐R. Ella‐Menye , C. Irvin , B. D. Ratner , S. Jiang , Nat. Biotechnol. 2013, 31, 553. 23666011 10.1038/nbt.2580

[107] X. Lin , P. Jain , K. Wu , D. Hong , H.‐C. Hung , M. B. O'Kelly , B. Li , P. Zhang , Z. Yuan , S. Jiang , Langmuir 2019, 35, 1544. 30265550 10.1021/acs.langmuir.8b02540 PMC6501560

[108] S. Chen , L. Li , C. Zhao , J. Zheng , Polymer 2010, 51, 5283.

[109] A. Sinclair , M. B. O'Kelly , T. Bai , H.‐C. Hung , P. Jain , S. Jiang , Adv. Mater. 2018, 30, 1803087. 10.1002/adma.201803087 PMC6588167 30066374

[110] P. Zhang , F. Sun , C. Tsao , S. Liu , P. Jain , A. Sinclair , H.‐C. Hung , T. Bai , K. Wu , S. Jiang , Proc. Natl. Acad. Sci. U. S. A. 2015, 112, 12046. 26371311 10.1073/pnas.1512465112 PMC4593115

[111] Q. Shao , S. Jiang , J. Phys. Chem. B 2013, 117, 1357. 23316760 10.1021/jp3094534

[112] S. P. Nikam , P. Chen , K. Nettleton , Y.‐H. Hsu , M. L. Becker , Biomacromolecules 2020, 21, 2714. 32459090 10.1021/acs.biomac.0c00456

[113] C. Englert , M. Pröhl , J. A. Czaplewska , C. Fritzsche , E. Preußger , U. S. Schubert , A. Traeger , M. Gottschaldt , Macromol. Biosci. 2017, 17, 1600502. 10.1002/mabi.201600502 28371343

[114] C. Englert , A.‐K. Trützschler , M. Raasch , T. Bus , P. Borchers , A. S. Mosig , A. Traeger , U. S. Schubert , J. Controlled Release 2016, 241, 1. 10.1016/j.jconrel.2016.08.039 27586188

[115] B. Sun , W. Hong , E. S. Thibau , H. Aziz , Z.‐H. Lu , Y. Li , ACS Appl. Mater. Interfaces 2015, 7, 18662. 26244847 10.1021/acsami.5b05097

[116] H. Kang , S. Jung , S. Jeong , G. Kim , K. Lee , Nat. Commun. 2015, 6, 6503. 25790133 10.1038/ncomms7503 PMC4382999

[117] L. Yan , Y. Song , Y. Zhou , B. Song , Y. Li , Org. Electron. 2015, 17, 94.

[118] Y. Zhou , C. Fuentes‐Hernandez , T. M. Khan , J.‐C. Liu , J. Hsu , J. W. Shim , A. Dindar , J. P. Youngblood , R. J. Moon , B. Kippelen , Sci. Rep. 2013, 3, 1536. 23524333 10.1038/srep01536 PMC3607174

[119] Y. Guo , X. Zhou , Q. Tang , H. Bao , G. Wang , P. Saha , J. Mater. Chem. A 2016, 4, 8769.

[120] J. Kang , D. Son , G.‐J. N. Wang , Y. Liu , J. Lopez , Y. Kim , J. Y. Oh , T. Katsumata , J. Mun , Y. Lee , L. Jin , J. B.‐H. Tok , Z. Bao , Adv. Mater. 2018, 30, 1706846. 10.1002/adma.201706846 29424026

[121] H. Liao , S. Liao , X. Tao , C. Liu , Y. Wang , J. Mater. Chem. C 2018, 6, 12992.

[122] Q. Yan , M. Zhou , H. Fu , J. Mater. Chem. C 2020, 8, 7772.

[123] Z. Chen , M. Yang , Q. Shi , X. Kuang , H. J. Qi , T. Wang , Sci. Rep. 2019, 9, 17902. 31784554 10.1038/s41598-019-54045-w PMC6884634

[124] C. Hou , Z. Xu , W. Qiu , R. Wu , Y. Wang , Q. Xu , X. Y. Liu , W. Guo , Small 2019, 15, 1805084. 10.1002/smll.201805084 30690886

[125] X. Ji , L. Song , S. Zhong , Y. Jiang , K. G. Lim , C. Wang , R. Zhao , J. Phys. Chem. C 2018, 122, 16909.

[126] R. K. Pal , S. C. Kundu , V. K. Yadavalli , ACS Appl. Mater. Interfaces 2018, 10, 9620. 29480009 10.1021/acsami.7b19309

[127] Y. H. Jung , T.‐H. Chang , H. Zhang , C. Yao , Q. Zheng , V. W. Yang , H. Mi , M. Kim , S. J. Cho , D.‐W. Park , H. Jiang , J. Lee , Y. Qiu , W. Zhou , Z. Cai , S. Gong , Z. Ma , Nat. Commun. 2015, 6, 7170. 26006731 10.1038/ncomms8170 PMC4455139

[128] W. T. Gao , L. Q. Zhu , H. Xiao , F. Yu , J. Tao , D. Y. Wan , J. M. Zhou , Org. Electron. 2018, 56, 82.

[129] J. Lee , H. R. Cho , G. D. Cha , H. Seo , S. Lee , C.‐K. Park , J. W. Kim , S. Qiao , L. Wang , D. Kang , T. Kang , T. Ichikawa , J. Kim , H. Lee , W. Lee , S. Kim , S.‐T. Lee , N. Lu , T. Hyeon , S. H. Choi , D.‐H. Kim , Nat. Commun. 2019, 10, 5205. 31729383 10.1038/s41467-019-13198-y PMC6858362

[130] L. Teng , S. Ye , S. Handschuh‐Wang , X. Zhou , T. Gan , X. Zhou , Adv. Funct. Mater. 2019, 29, 1808739.

[131] E. J. Curry , K. Ke , M. T. Chorsi , K. S. Wrobel , A. N. Miller , A. Patel , I. Kim , J. Feng , L. Yue , Q. Wu , C.‐L. Kuo , K. W.‐H. Lo , C. T. Laurencin , H. Ilies , P. K. Purohit , T. D. Nguyen , Proc. Natl. Acad. Sci. U. S. A. 2018, 115, 909. 29339509 10.1073/pnas.1710874115 PMC5798324

[132] J. McGinness , P. Corry , P. Proctor , Science 1974, 183, 853. 4359339 10.1126/science.183.4127.853

[133] A. Napolitano , A. Pezzella , M. d'Ischia , G. Prota , Tetrahedron 1996, 52, 8775.

[134] C. M. Boutry , Y. Kaizawa , B. C. Schroeder , A. Chortos , A. Legrand , Z. Wang , J. Chang , P. Fox , Z. Bao , Nat. Electron. 2018, 1, 314.

[135] C. M. Boutry , L. Beker , Y. Kaizawa , C. Vassos , H. Tran , A. C. Hinckley , R. Pfattner , S. Niu , J. Li , J. Claverie , Z. Wang , J. Chang , P. M. Fox , Z. Bao , Nat. Biomed. Eng. 2019, 3, 47. 30932072 10.1038/s41551-018-0336-5

[136] W. Li , Q. Liu , Y. Zhang , C. Li , Z. He , W. C. H. Choy , P. J. Low , P. Sonar , A. K. K. Kyaw , Adv. Mater. 2020, 32, 2001591. 10.1002/adma.202001591 32584502

[137] M. J. Tan , C. Owh , P. L. Chee , A. K. K. Kyaw , D. Kai , X. J. Loh , J. Mater. Chem. C 2016, 4, 5531.

[138] M. Seck , N. Mohammadian , A. K. Diallo , S. Faraji , M. Erouel , N. Bouguila , D. Ndiaye , K. Khirouni , L. A. Majewski , Org. Electron. 2020, 83, 105735.

[139] E. Shin , J. Yoo , G. Yoo , Y.‐J. Kim , Y. S. Kim , Chem. Eng. J. 2019, 358, 170.

[140] F. Sugiyama , A. T. Kleinschmidt , L. V. Kayser , M. A. Alkhadra , J. M.‐H. Wan , A. S.‐C. Chiang , D. Rodriquez , S. E. Root , S. Savagatrup , D. J. Lipomi , Macromolecules 2018, 51, 5944. 30930487 10.1021/acs.macromol.8b00846 PMC6435287

[141] H. Tran , V. R. Feig , K. Liu , Y. Zheng , Z. Bao , Macromolecules 2019, 52, 3965.

[142] J. Borges‐González , C. J. Kousseff , C. B. Nielsen , J. Mater. Chem. C 2019, 7, 1111.

[143] D. T. Simon , E. O. Gabrielsson , K. Tybrandt , M. Berggren , Chem. Rev. 2016, 116, 13009. 27367172 10.1021/acs.chemrev.6b00146

[144] K. Feron , R. Lim , C. Sherwood , A. Keynes , A. Brichta , P. C. Dastoor , Int. J. Mol. Sci. 2018, 19, 2382. 10.3390/ijms19082382 PMC6121695 30104515

[145] J. H. Lee , H. Kim , J. H. Kim , S.‐H. Lee , Lab Chip 2016, 16, 959.26891410

[146] A. Srinivasan , M. Tahilramani , J. T. Bentley , R. K. Gore , D. C. Millard , V. J. Mukhatyar , A. Joseph , A. S. Haque , G. B. Stanley , A. W. English , R. V. Bellamkonda , Biomaterials 2015, 41, 151. 25522974 10.1016/j.biomaterials.2014.11.035 PMC4324612

[147] S. M. Lee , H. J. Byeon , B. H. Kim , J. Lee , J. Y. Jeong , J. H. Lee , J.‐H. Moon , C. Park , H. Choi , S.‐H. Lee , K.‐H. Lee , BioChip J. 2017, 11, 153.

[148] M. Du , S. Guan , L. Gao , S. Lv , S. Yang , J. Shi , J. Wang , H. Li , Y. Fang , Small 2019, 15, 1900582. 10.1002/smll.201900582 30977967

[149] M. J. Donahue , A. Sanchez‐Sanchez , S. Inal , J. Qu , R. M. Owens , D. Mecerreyes , G. G. Malliaras , D. C. Martin , Mater. Sci. Eng., R 2020, 140, 100546.

[150] M. Di Lauro , S. Benaglia , M. Berto , C. A. Bortolotti , M. Zoli , F. Biscarini , Colloids Surf., B 2018, 168, 143. 10.1016/j.colsurfb.2018.03.022 29588094

[151] A. Campana , T. Cramer , D. T. Simon , M. Berggren , F. Biscarini , Adv. Mater. 2014, 26, 3874. 24644020 10.1002/adma.201400263

[152] W. Lee , D. Kim , N. Matsuhisa , M. Nagase , M. Sekino , G. G. Malliaras , T. Yokota , T. Someya , Proc. Natl. Acad. Sci. U. S. A. 2017, 114, 10554. 28923928 10.1073/pnas.1703886114 PMC5635873

[153] T. Ware , D. Simon , K. Hearon , C. Liu , S. Shah , J. Reeder , N. Khodaparast , M. P. Kilgard , D. J. Maitland , R. L. Rennaker , W. E. Voit , Macromol. Mater. Eng. 2012, 297, 1193. 25530708 10.1002/mame.201200241 PMC4268152

[154] D.‐H. Do , M. Ecker , W. E. Voit , ACS Omega 2017, 2, 4604. 30023725 10.1021/acsomega.7b00834 PMC6044618

[155] S. Park , S. W. Heo , W. Lee , D. Inoue , Z. Jiang , K. Yu , H. Jinno , D. Hashizume , M. Sekino , T. Yokota , K. Fukuda , K. Tajima , T. Someya , Nature 2018, 561, 516. 30258137 10.1038/s41586-018-0536-x

[156] X. Strakosas , M. Bongo , R. M. Owens , J. Appl. Polym. Sci. 2015, 132, 41735.

[157] G. E. Bonacchini , C. Bossio , F. Greco , V. Mattoli , Y.‐H. Kim , G. Lanzani , M. Caironi , Adv. Mater. 2018, 30, 1706091. 10.1002/adma.201706091 29460421

[158] C. Steiger , A. Abramson , P. Nadeau , A. P. Chandrakasan , R. Langer , G. Traverso , Nat. Rev. Mater. 2019, 4, 83. 10.1038/s41578-018-0070-3 PMC12393167 40895645

[159] S. Liu , Y. Fu , G. Li , L. Li , H. K. Law , X. Chen , F. Yan , Adv. Mater. 2017, 29, 1701733. 10.1002/adma.201701733 28707332

[160] A. Kyndiah , F. Leonardi , C. Tarantino , T. Cramer , R. Millan‐Solsona , E. Garreta , N. Montserrat , M. Mas‐Torrent , G. Gomila , Biosens. Bioelectron. 2020, 150, 111844. 31740253 10.1016/j.bios.2019.111844

